# CytofIn enables integrated analysis of public mass cytometry datasets using generalized anchors

**DOI:** 10.1038/s41467-022-28484-5

**Published:** 2022-02-17

**Authors:** Yu-Chen Lo, Timothy J. Keyes, Astraea Jager, Jolanda Sarno, Pablo Domizi, Ravindra Majeti, Kathleen M. Sakamoto, Norman Lacayo, Charles G. Mullighan, Jeffrey Waters, Bita Sahaf, Sean C. Bendall, Kara L. Davis

**Affiliations:** 1 Department of Pediatrics, Stanford University School of Medicine, Stanford, CA USA; 2 Medical Scientist Training Program, Stanford University School of Medicine, Stanford, CA USA; 3 Department of Medicine, Stanford University School of Medicine, Stanford, CA USA; 4 Department of Pathology, St. Jude Children’s Research Hospital, Memphis, TN USA; 5 Center for Cancer Cellular Therapy, Cancer Correlative Sciences Unit, Stanford University School of Medicine, Stanford, CA USA; 6 Department of Pathology, Stanford University School of Medicine, Stanford, CA USA

**Keywords:** Acute lymphocytic leukaemia, Software, Data integration

## Abstract

The increasing use of mass cytometry for analyzing clinical samples offers the possibility to perform comparative analyses across public datasets. However, challenges in batch normalization and data integration limit the comparison of datasets not intended to be analyzed together. Here, we present a data integration strategy, CytofIn, using generalized anchors to integrate mass cytometry datasets from the public domain. We show that low-variance controls, such as healthy samples and stable channels, are inherently homogeneous, robust against stimulation, and can serve as generalized anchors for batch correction. Single-cell quantification comparing mass cytometry data from 989 leukemia files pre- and post normalization with CytofIn demonstrates effective batch correction while recapitulating the gold-standard bead normalization. CytofIn integration of public cancer datasets enabled the comparison of immune features across histologies and treatments. We demonstrate the ability to integrate public datasets without necessitating identical control samples or bead standards for fast and robust analysis using CytofIn.

## Abstract (web summary)

Challenges in batch normalization and data integration limit the comparison of existing mass cytometry datasets. Here, the authors report CytofIn that can integrate mass cytometry datasets from the public domain and reveal cellular features associated with immune oncology by analyzing five public cancer datasets.

## Introduction

Mass cytometry (cytometry time of flight or CyTOF) is an increasingly widespread technique for the discovery and monitoring of cell populations using single-cell, high-parameter protein measurements^1^. Mass cytometry offers the ability to analyze millions of cells quickly and inexpensively compared to single-cell genomic platforms yet can be combined with approaches like single-cell RNA sequencing for the complimentary analysis of cell populations^2^. The utility of mass cytometry has been demonstrated in numerous studies relating to immune response, cancer, and healthy tissue development^3–8^. Furthermore, mass cytometry is being widely integrated into correlative studies in clinical trials such as in the Cancer Immune Monitoring and Analysis Centers (CIMAC) and the Partnership for Accelerating Cancer Therapies (PACT) initiatives, where it has been used to determine immune correlatives associated with clinical outcomes in cancer immunotherapies^9^. The rapid growth in the applicability of mass cytometry for clinical measurement also led to an increased availability of mass cytometry data in the public domain. Platforms such as FlowRepository and Cytobank support storage, annotation, and sharing of flow and mass cytometry datasets^10,11^. Consequently, integrating datasets from different studies for comparative analysis emerges as a desired approach that could lead to unexpected discoveries not afforded by individual studies.

**Fig. 1:**
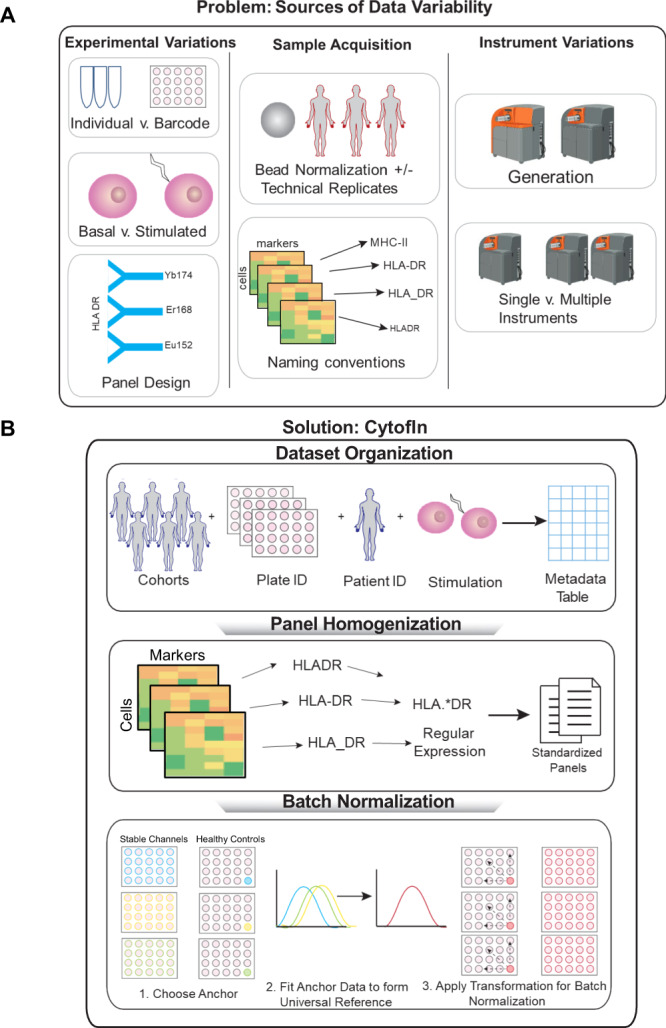
Overview of CytofIn for mass cytometry data integration. **A** The challenge of mass cytometry dataset integration from the public domain is limited by sources of data variability during experimental procedures, sample acquisition, and instrument variations, resulting in datasets with heterogeneous files and batch effects. **B** CytofIn, a computational pipeline for integrated analysis of mass cytometry data from the public domain. First, CytofIn organizes mass cytometry data by extracting sample information and experimental conditions and summarizes using a metatable for batch processing. Using regular expression matching, CytofIn homogenizes mass cytometry data files by identifying common text patterns found in heterogeneous sets of channel labels. Finally, CytofIn normalizes mass cytometry datasets using generalized anchors, which are non-identical references that exhibit low signal variability across experiments, eliminating the need for beads or identical technical replicates. Figure created with Biorender.

Batch effects remain a major limiting factor when comparing mass cytometry datasets. In this case, biological signals can be confounded by technical noise that is irrelevant to biological sources, making data interpretation and inference challenging. Batch effects arise from multiple factors during the experimental procedures including differential sensitivity across cytometers, metal sensitivity and oxidation, antibody variations, and channel spillover^12^ (Fig. 1A). Batch effects can be minimized by standardized experimental protocols where sources of variation are systematically reduced, including consistent sample preparation and staining, careful control of antibody reagents, and consistent instrument setup^13,14^. Still, some batch variability will always exist between CyTOF experiments even when highly standardized workflows are followed^15^.

Within individual studies, batch effects are commonly addressed using bead normalization^16^. Bead normalization uses metal labeled polystyrene beads added to the cell suspension to correct for signal fluctuations during data acquisition^16^. Leipold et al. demonstrated that bead normalization alone achieved a < 30% coefficient of variation (CV) of median signal intensity when comparing healthy human peripheral blood mononuclear cells (PBMCs) analyzed at six different centers^17^. In this study, all PBMC samples were prepared at a single center a priori, therefore removing one common source of variability. Since bead information is not prevalently shared between experiments conducted at different centers, public datasets lacking bead data often cannot be normalized with new datasets. In addition, bead data only corrects for unimodal instrument sensitivity differences across channels.

Recently, several normalization approaches have been proposed that obviate the need for bead standards. Methods like CyTofRUV and CytoNorm include identical technical replicates, (aliquots of the same sample) in each batch to correct data distributions of protein signals based on a goal distribution^18–20^. However, the lack of identical replicates across existing datasets remains a major limitation to applying these methods for the analysis of public datasets or datasets collected over time^19^. Non-anchor-based normalization methods like quantile normalization estimate a reference distribution based on the average of each quantile across all samples under the assumption that the statistical distributions of all samples are identical^21^. Although identical controls are not required when using quantile normalization, the reference distribution needs to be re-estimated with the addition of new samples and the assumption that all samples are identically distributed can remove features of biological importance as well as introduce artificial bias^22^. Thus, a method to enable cross-dataset comparison without identical technical replicates is needed (Supplementary Table 1)^19^.

File heterogeneity between datasets is common when different naming conventions are adopted for antibodies or antibodies are labeled on different metals between panels. Homogenizing such heterogeneous files is often the first step to enable comparative analyses yet is currently unwieldy for large datasets. Panel homogenization refers to the process of aligning the antigen panels across multiple CyTOF experiments by removing channels not shared across cohort samples and standardizing the antigen name used in each channel. When comparing mass cytometry data across independent sources, multiple panels will need to be homogenized simultaneously. Although programs like Fluidigm and Premessa (https://github.com/ParkerICI/premessa) enable manual editing of panel labels, preprocessing panels from large cohorts is prohibitively time-consuming and therefore necessitates automation.

Here we present CytofIn (CyTOF Integration), a computational pipeline for integrating mass cytometry data from the public domain using generalized anchors, which include healthy control samples and stable channels that exhibit low signal variability across datasets and can be used as an approximation of identical anchors (Fig. 1B). Using data from 989 leukemia patient samples and a small subset of lymphoma patient samples, we demonstrate the utility of CytofIn for integrating and comparing datasets across instruments or over time without necessitating identical control samples or bead standards. Application of CytofIn to five cancer datasets from the public domain enables comparison of infiltrating immune cells across cancer histologies and identifies immune features associated with immune checkpoint inhibition therapies.

## Results

### Healthy controls as generalized anchors

**Fig. 2:**
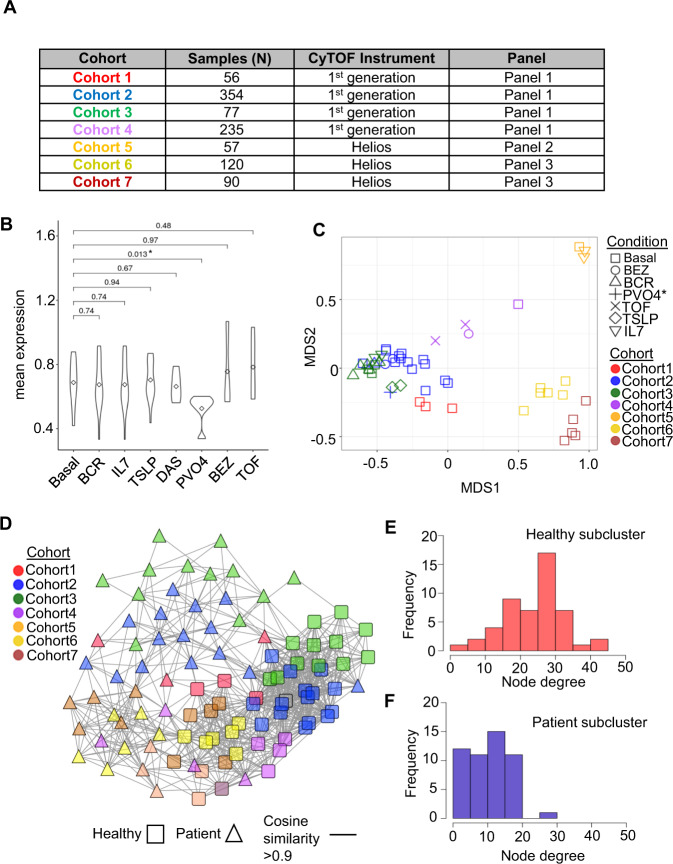
Characterization of healthy controls as generalized anchors. **A** Seven cohorts of B-lymphoblastic leukemia samples were collected during 2014–2019. The samples were collected on two different CyTOF instruments using three antibody panels with slight variations in naming conventions, the number of proteins measured, and protein-metal labels. **B** Effects of ex vivo perturbation with cytokines or small molecules on the mean expression of 36 consensus markers in the healthy control samples. The significance of the overlap in the distribution was quantified by *P*-values using a two-sided Wilcoxon test across 36 protein markers comparing basal v. perturbation conditions. The perturbation conditions include B-cell receptor cross-linking (BCR), Dasatinib (DAS), thymic stromal lymphopoietin (TSLP), BEZ-235 (BEZ), sodium orthovanadate (PVO4), tofacitinib (TOF), and IL-7 (IL7). **C** Multidimensional analysis of the 50 healthy controls from 7 cohorts. Note that shapes represent conditions and colors represent cohorts. **D** Unsupervised clustering of both healthy and patient samples using an expression similarity network where nodes represent samples and edges represent the cosine similarity between sample mean expressions. The healthy samples form a highly connected subcluster distinctly separated from that of the patient samples using a stringent similarity threshold of 0.9. Comparison of node degree (number of connected edges) distribution between (**E**) healthy (red) and (**F**) patient subclusters (blue). The healthy subcluster exhibited a higher degree of network connectivity (node degree_avg_ = 24.4) to the patient subcluster (node degree_avg_ = 10.9), as revealed by denser between-node edges, indicating smaller within-sample variations. Source data are provided as a Source Data file.

Healthy controls are potential generalized anchors for batch normalization due to their low individual variability as well as their wide availability from clinical experiments. We first evaluated the within- and between-cohort variability using seven cohorts of acute lymphoblastic leukemia (ALL) samples collected over 6 years in our laboratory. Within each cohort, a healthy bone marrow or peripheral blood control was included in each barcode plate for a total of 989 leukemia samples distributed across 50 plates. The ALL samples were analyzed using three antibody panels on two generations of mass cytometers where each antibody panel had slight variations in naming conventions, the number of proteins measured, and the protein-metal label (Fig. 2A and Supplementary Table 2). Here, the original data refers to data files that underwent bead normalization within each cohort to account for instrument-related signal decay but no further batch normalization was performed (see Methods). In addition, we use the term bead normalization for batch normalization using bead signals across cohorts (in addition to within cohorts).

To facilitate cross-dataset analysis, we developed a computational pipeline for automated panel homogenization of leukemia samples across the datasets. To enable batch preprocessing, a metadata table consisting of subfields including cohort names, plate numbers, patient identifiers, and perturbation conditions was used to annotate the datasets (Supplementary Data 1). Next, regular expression searches were used to capture common text patterns found in channel labels among 989 CyTOF files to identify non-identical, synonymous terms. These files were then automatically homogenized to a single panel of 36 consensus markers (Fig. 1B and Supplementary Data 2)^23^.

Since some of the healthy control samples had undergone ex vivo perturbation with cytokines or small molecule inhibitors, we evaluated the impact of perturbation on protein expression across the healthy controls. Except for treatment with the phosphatase inhibitor sodium orthovanadate (PVO_4_), we did not observe significant differences in the overlap in distributions of the mean protein expression in the stimulated or basal condition across the 36 consensus markers (Fig. 2B and Supplementary Fig. 1). Visualization of the data using multidimensional scaling (MDS) based on the mean expression profiles demonstrated that healthy controls were, in fact, strongly segregated by cohort rather than the stimulation condition, suggesting that the major source of variation between healthy samples was batch effects (Fig. 2C).

We next evaluated the variability of healthy controls compared to the patient samples to assess their robustness as generalized anchors. Ideally, signal variability between healthy controls should be small so that adjustments after normalization can be attributed to batch effects. Indeed, network-based similarity clustering of mean expression profiles from both healthy controls and selected leukemia samples matched by stimulation condition resulted in distinct healthy and patient subclusters (Fig. 2D)^24^. Comparing the network connectivity of these distinct subclusters, the healthy subcluster exhibited a higher level of network connectivity (node degree_avg_ = 24.4) than the patient subcluster (node degree_avg_ = 10.9), as revealed by denser between-node edges, indicating smaller within-sample variations (Fig. 2E, F). A stronger cohort stratification in the healthy subcluster was consistent with the observed batch variations (Fig. 2C). Taken together, our analysis demonstrated that healthy controls exhibited low signal variability and their resistance to ex vivo stimulation made them potential generalized anchors for batch normalization.

### CytofIn batch normalization using healthy controls

**Fig. 3:**
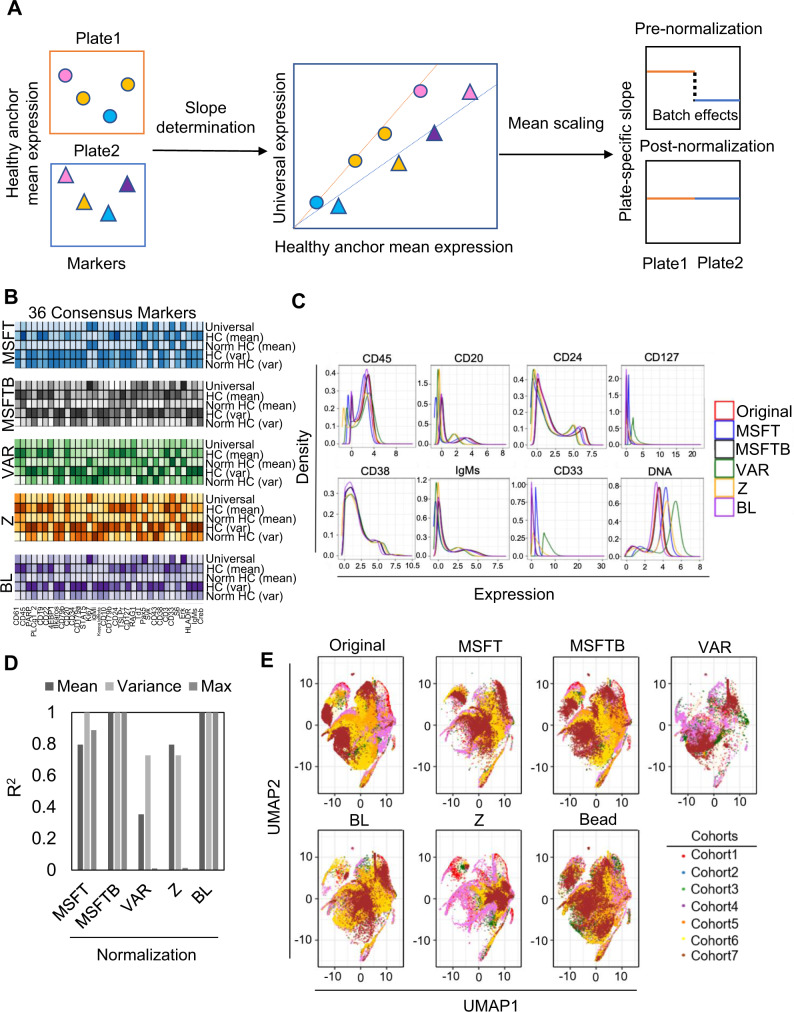
Batch normalization functions. **A** Batch normalization using bead-like (BL) normalization. Markers of healthy control were used to generate plate-specific slopes to fit the universal healthy reference data distribution. To minimize batch effects, mean expressions of protein markers from each sample on the plate were scaled according to each plate-specific slope as in the bead standardization procedure. **B** The effect of five normalization functions: meanshift (MSFT), meanshift bulk (MSFTB), variance (VAR), z-score (Z), and bead-like (BL) on the data distribution of one representative healthy control sample. The changes in mean expression and variance values of 36 consensus markers were visualized using heatmaps where the colors were correlated to signal intensity. **C** Density plot of 8 consensus protein markers from healthy samples (*n* = 3) pre- and post-batch normalization using each five normalization functions (See Supplementary Fig. 3 for the distribution of all 36 consensus markers). **D** Correlation analysis between bead and batch normalized signals using each of the five normalization functions assessed by mean expression, variance values, and peak intensity: MSFT, MSFTB, VAR, Z, and BL normalizations. **E** Visualization of batch effects in 50 healthy anchors normalized by each five normalization functions using UMAP. Note that points represent single cells and were colored according to respective cohorts. Source data are provided as a Source Data file.

Given that the healthy controls in leukemia cohorts were robust to ex vivo stimulation and exhibited low signal variability, we proceeded to utilize them as generalized anchors for batch normalization. We evaluated several normalization functions to fit the generalized anchors to a goal distribution based on central tendency and dispersion of the 36 consensus markers (Fig. 1B and Methods). The healthy control files from the leukemia cohorts (*n* = 50) were aggregated by concatenating the mean expression of 36 consensus channels from all cells to define a reference distribution, referred to as universal reference. To select healthy anchors from each plate, we prioritized healthy samples in basal over stimulation conditions or stimulation conditions that have minimal effect on the expression. The data distribution of each healthy anchor was adjusted to the universal reference distribution using normalization functions (see Methods). The same adjustment was then used to normalize patient samples in groups per barcode plate (Fig. 1B). We assessed the effect of four well-characterized normalization functions: meanshift (MSFT), meanshift bulk (MSFTB), variance (VAR), and z-score (Z) to batch-normalize original mass cytometry data from the seven leukemia cohorts (see Methods)^25^. In addition, we proposed a bead-like (BL) normalization function that scales original mean expression based on the slope of a best-fit regression curve between healthy and universal reference expression across all channels, similar to that performed by the bead standardization procedure (Fig. 3A)^16^. Finally, the cohorts were also batch normalized using bead normalization for comparison. The expression and variance values of 36 markers from one representative healthy control pre- and post-normalization using each normalization function are shown in Fig. 3B.

To identify the normalization function that best preserved the original data distribution of each channel, we first quantified the changes in mean expression, variance, and peak intensity of healthy samples from the raw signal post batch normalization. Expression profiling of 36 consensus markers using 1-dimensional density plots showed that MSFT, MSFTB, and BL normalizations had a minimal effect on raw protein marker distributions (Fig. 3C, Supplementary Fig. 2 and Supplementary Table 5). By contrast, larger fluctuations in peak intensity and variance values were observed using the Z or VAR normalizations, particularly in several lineage markers like CD45, CD20, CD24, and CD33 (Fig. 3C, Supplementary Fig. 2 and Supplementary Table 5). Consistently, data normalized using the MSFTB and BL normalization functions attained the highest correlation to the original signals compared to MSFT, VAR or Z normalizations based on each of the three parameters (Fig. 3D and Supplementary Fig. 3).

To further assess the degree of batch effect reduction by each normalization function, we sub-sampled 2000 cells from three representative healthy control samples per cohort among 7 cohorts and compared their single-cell distribution pre and post batch normalization using Uniform Manifold Approximation and Projection (UMAP) by projecting onto the same embedded space of the original samples (Fig. 3E and Methods)^26,27^. As expected, a strong batch effect was observed in the original samples while in the bead normalized samples, the batch effects were reduced (Fig. 3E and Supplementary Fig. 4). We saw various degrees of batch effect reduction using each of the five normalization functions (Fig. 3E and Supplementary Fig. 4). To quantify how each normalization function altered the distance between cells within and between cohorts, we compared the average cohort cell-to-cell Euclidian distance between the original and the batch normalized data (Supplementary Fig. 5). Overall, all normalization functions resulted in decreased intra and inter cohort distances except variance and z-score normalization functions (Supplementary Fig. 5). Compared to the original data, the average cell-cell distances between and within cohorts became closer after our CytofIn normalization procedure. Interestingly, MSFTB normalization attained the most similar cell-cell distance changes to that by the bead normalization.

We compared our batch normalization approach to two existing mass cytometry normalization methods, CytoNorm (CN) and CytofRUV (CV), and Seurat (ST), a batch correction method for scRNA-seq (Supplementary Figs. 6 and 7)^18,28,29^. CN and CV rely on identical anchors, thus we made the assumption that the healthy anchors from each batch are approximately identical due to their low variance. We sampled 10% of the cells (200 cells per cohort) and compared performance using two benchmarking metrics kBET and LISI, which measure local batch and cell mixing effects by comparing the local and global label distribution on the UMAP space (Supplementary Figs. 6A, 7A and Methods)^30–32^. Here, a low kBET index or a high LISI index were correlated to increased label mixing. In addition, we included the original and bead normalized samples as negative and positive controls.

Our benchmark study showed that MSFTB, MSFT, and BL normalization performed favorably in terms of batch mixing based on the difference between their kBET and LISI indexes compared to bead normalization (Supplementary Fig. 6C, D). Importantly, there were minimal changes in cell distribution of data normalized by MSFTB in contrast to CV or ST batch normalization although CV indicated a higher level of batch mixing (Supplementary Fig. 6A). To evaluate CytofIn’s ability to maintain cell types after batch normalization, we performed FlowSOM clustering (100 clusters) on batch normalized healthy samples (Supplementary Fig. 7A)^33^. Except for CV, all methods were similar to bead normalized data based on the kBET index, possibly due to the small size of each cell cluster. MSFTB, CN, and ST performed similarly to bead normalization based on the LISI index (Supplementary Fig. 7B, C). Overall, our analyses demonstrated that MSFTB and BL normalization performed favorably to the gold standard bead normalization enabling healthy control samples to be used as generalized anchors for batch normalization going forward.

### CytofIn replicates bead normalization on patient leukemia samples

Bead normalization is currently the gold standard for reducing batch effects across datasets provided that the bead information is available. We next evaluated the ability of CytofIn to replicate the performance of bead normalization using the full leukemia cohorts. To compare the performance of batch normalization using the MSFTB and BL normalization functions to the bead standardization, the leukemia cohorts were separated into two batches: Batch A (cohort 1–4) obtained on a CyTOF 1 cytometer and Batch B (cohort 5–7) obtained on a Helios cytometer, where these two cytometers exhibited differences in sensitivity for signal readout (Supplementary Data 1). To determine the mean expression difference between the batch and bead normalized signals across 36 consensus markers, bead normalization was first performed as is standard by normalizing Batch A and Batch B together prior to debarcoding (Supplementary Fig. 8, left). In parallel, each batch was independently bead normalized and then batch normalized using each of the five normalization functions for comparison (Supplementary Fig. 8, right).

**Fig. 4:**
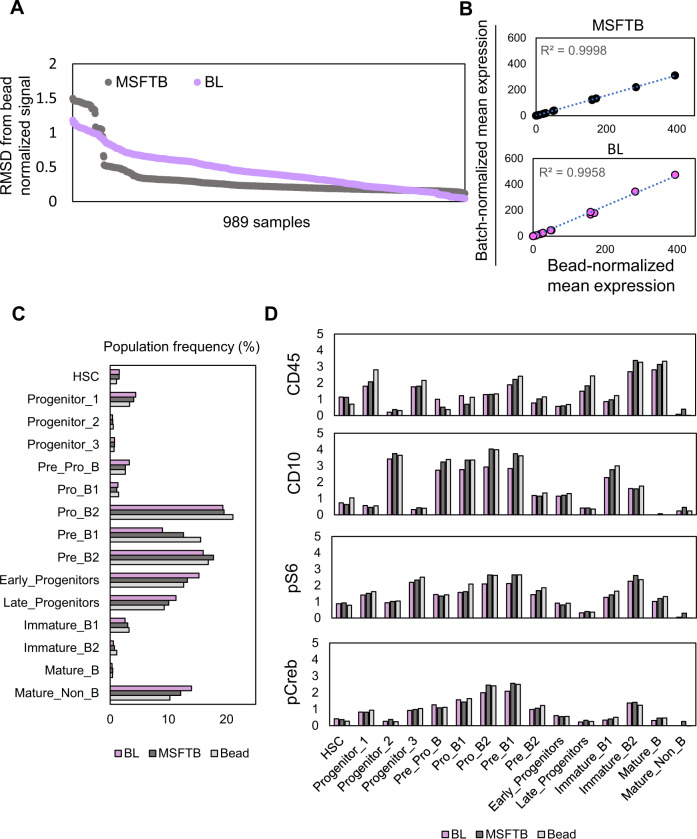
Batch normalization recapitulates bead normalization performance. **A** Comparison of batch and bead normalized mean expression across 989 leukemia samples using MSFTB and BL normalizations. The differences between batch and bead normalized signals were quantified by the RMSD values. **B** Correlation analysis between batch and bead normalized expression of 36 consensus marker expressions from one selected patient sample. The R^2^ values normalized using MSFTB and BL normalizations were 0.999, and 0.996, respectively. **C** Comparison of 15 ALL subpopulation frequencies in 50 selected leukemia patient samples normalized by MSFTB and BL normalization functions compared to bead standardization (see Supplementary Fig. 10 for the complete quantification of 15 subpopulation frequencies normalized by each normalization method). **D** Mean expression of four markers: CD45, CD10, pS6, and pCreb in 15 subpopulations of 50 selected leukemia patient samples normalized using MSFTB, BL, or bead normalization (see Supplementary Fig. 11 for the complete quantification on the mean expression of four markers by each of the five normalization functions). Source data are provided as a Source Data file.

The differences between batch and bead normalized mean expression of 36 consensus markers from 989 samples were quantified by root-mean-square-deviation (RMSD) values (Supplementary Fig. 9A). Batch correction using MSFTB achieved the highest consistency to that by bead normalization with an average RMSD value of 0.33 (Fig. 4A and Supplementary Fig. 9B). BL performed reasonably well with an average RMSD value of 0.45 while MSFT, VAR, or Z normalizations all resulted in RMSD values of > 0.5 (0.6–1.33) (Fig. 4A and Supplementary Fig. 9B). Likewise, both MSFTB and BL-normalized signals achieved strong correlations (R^2^ > 0.99) to bead normalized signals (Fig. 4B). One important assumption for our approach is that the variance between generalized anchors should be a fair degree smaller than that between target samples in order for batch effects to be accurately estimated and corrected. Indeed, by correlating the RMSD values between each plate-specific healthy anchor to the universal healthy reference, we saw that the normalized samples that have the strongest deviation from the bead normalization result e.g., cohort 6 in our data, are the ones with the highest RMSD values and vice versa (Supplementary Fig. 10A). We further evaluated the correlation between the deviation of the healthy anchor mean expression from each plate (batch) to the universal healthy reference using each normalization function and then assessed the accuracy of the batch normalization result (measured using the deviation from the bead normalization result). We showed that the amount of deviation from the aggregated universal reference was indeed correlated to the normalization performance for all CytofIn normalization methods (Supplementary Fig. 10B).

Critical downstream analyses of mass cytometry data depend on the identification of cell populations and their features. To this end, we quantified the population abundance and mean expression of the 36 consensus markers among 15 subpopulations previously characterized in B-cell ALL (Supplementary Fig. 11A)^6^. To enable controlled comparison, the gating parameters for each subpopulation were optimized by gating on the concatenated healthy samples and the same parameters were applied to classify pre and post-normalized samples (Supplementary Fig. 11A). By evaluating the difference (Δmean ± s.d.) between batch and bead normalized subpopulation frequencies across 15 subpopulations, we showed that, on average, MSFTB normalization was best at capturing bead standardized subpopulation abundances than MSFT, BL, VAR, or Z normalizations (Fig. 4C, Supplementary Fig. 12). Among the classified subpopulations, the ProB2 and PreB1 cells were most affected by the batch normalization procedure as these two subpopulations were defined by a similar set of protein markers (Fig. 4C and Supplementary Fig. 11A). For each subpopulation, we further examined the mean signal intensity of four selected proteins: CD10, CD45, pS6 and pCreb not used to gate the developmental populations. As expected, the expression differences (Δmean ± s.d.) of these 4 markers from the bead standardized expression were minimal using MSFTB and BL normalizations compared to MSFT, VAR, or Z normalizations (Fig. 4D, Supplementary Fig. 13 and Supplementary Table 6). To determine if MSFTB and BL normalizations alter data distribution at single-cell resolution, we compared the biaxial plot of CD45-CD10 and pS6-pCreb of the ProB2 subpopulation (Supplementary Fig. 11B). Our analyses showed that the MSFTB normalization had minimal effect on the single-cell data distributions and that the adjustment was similar to that made by bead standardization (Supplementary Fig. 11B). On the other hand, the reduction in contour size by BL normalization may likely be due to mean scaling that minimizes the data variance and a similar effect was also observed in the BL batch normalized healthy anchor expression (Fig. 3C and Supplementary Fig. 5). Still, the RMSD values of mean marker expression of BL to that by bead normalization were the second-lowest following MSFTB (Supplementary Fig. 9A, B). Overall, our analysis showed that MSFTB and BL normalization can faithfully recapitulate the bead normalized signal in 7 leukemia cohorts with similar effects on subpopulation abundances and features.

### Low variance channels as generalized anchors for batch normalization

**Fig. 5:**
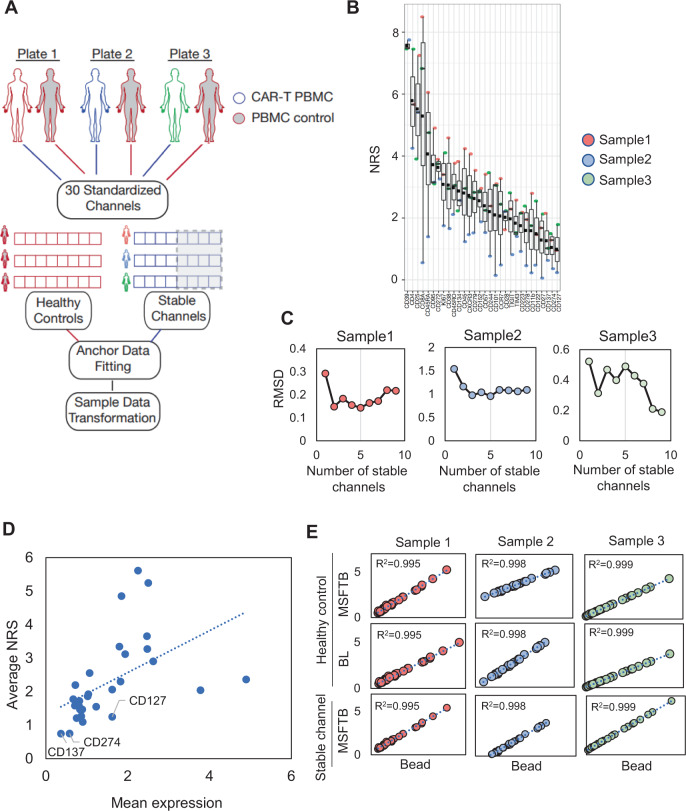
Evaluation of stable channels as generalized anchors for batch normalization. **A** The application of CyTOF data integration pipeline to standardize three patient PBMC samples from patients receiving CAR-T cell therapy. Batch normalization was performed using two types of generalized anchors: healthy peripheral blood mononuclear cell (PBMC) samples or stable channels. The generalized anchors were fitted to the universal reference data distribution followed by data normalization. **B** Identification of stable channels using a PCA-based non-redundancy score (NRS) to rank-order marker variability in three CAR-T patient samples (*n* = 3). The band indicates the median, the black square indicates the mean, the box indicates the first and third quartiles and the whiskers indicate ± 1.5 × interquartile range. Channels with NRS < 2 were identified. **C** To determine the optimal number of stable channels, the RMSD values between batch and bead normalized signals were determined by varying the number of stable channels for each sample. Minimum RMSD values were achieved with the top 3–4 stable channels. **D** Correlation between mean marker expression and channel stability measured by NRS values (R^2^~0.26). The top three stable channels: CD274, CD137, and CD127 are labeled. **E** Correlation between batch and bead normalization performance for three CAR-T cell samples using healthy control samples or top three stable channels as generalized anchors. Each dot represents the mean expression of 30 consensus markers normalized by bead (*x*-axis) or by batch normalization (*y*-axis). The correlation values were computed across 30 consensus markers for each sample. Batch normalization using stable channels normalized by either MSFTB or BL normalizations achieved comparable performance to that using external anchors with R^2^ values > 0.9. Source data are provided as a Source Data file.

Given that healthy controls may not be present in all datasets, we further explored the possibility of using low variance channels as generalized anchors for normalizing mass cytometry data (Fig. 5A). Similar to healthy control samples, we assume that channels with low signal variability are more likely to be invariant between samples in the same batch than channels with high signal variability; thus, their variations between batches can be used to estimate batch effects. To test the feasibility of this approach, we compared the performance of batch normalization using two types of generalized anchors—healthy controls or stable channels—to bead normalization on peripheral blood mononuclear cells (PBMCs) from three lymphoma patients undergoing CAR-T cell therapy (Fig. 5A). These samples were collected over 18 months in three batches in which one healthy PBMC control sample was included in each batch (Fig. 5A). Each patient sample was first homogenized to a panel of 30 consensus markers. All three samples were separately normalized using either healthy control as generalized anchors, stable channels as generalized anchors, or bead normalization for comparison. For batch normalization using healthy control samples, the mean expression of three healthy PBMC controls were aggregated to generate the universal reference and then batch normalized using either the MSFTB or BL normalization.

To identify suitable stable channels for batch normalization, we applied a PCA-based non-redundancy score (NRS) to rank-order the variability of each marker across the three patient samples and identify stable channels (Fig. 5B and Methods)^34^. To further determine the optimal number of stable channels for batch normalization, RMSD values were computed between the batch and bead normalized signals by varying the number of included stable channels. In two of the three samples, RMSD values reached a minimum when three to four stabilized channels were considered, which corresponds to NRS cutoff < 1 (Fig. 5C and Supplementary Fig. 14). Overall, the average RMSD values slowly decrease with increasing NRS after the first two stable channels (Supplementary Fig. 14). To determine whether stable channels were strictly limited to low expression markers, we compared the mean expression to NRS values of each marker but did not observe a correlation (R^2^ < 0.27) (Fig. 5D).

To batch-normalize the three lymphoma patient samples, the mean expression of the three most stable channels—CD127, CD274, and CD137—were aggregated to define the universal reference and subsequently normalized using the MSFTB normalization function. For validation, all three samples that were batch normalized using healthy controls achieved high correlations to bead normalized signal (R^2^ > 0.99) (Fig. 5E). Importantly, we showed that batch normalization using stable channels as generalized anchors was able to achieve comparable performance (R^2^ > 0.99) (Fig. 5E). We yielded similar kBET and LISI indexes when evaluating healthy sample data normalized by healthy control, stable channel or the two combined (Supplementary Fig. 15). Together, these results demonstrate that stable channels identified from shared markers across multiple CyTOF datasets may serve as robust generalized anchors for batch normalization. The flexibility of this approach enables batch normalization to be performed across mass cytometry datasets in the public domain.

### CytofIn dataset integration in the public domain

**Fig. 6:**
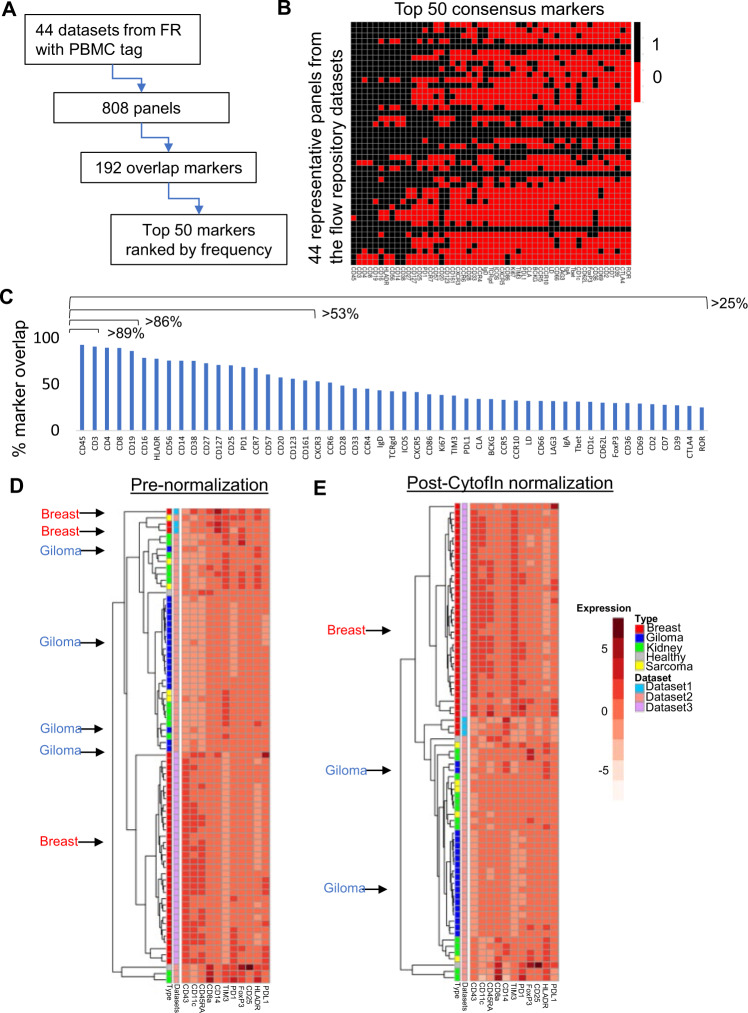
CytofIn integration of mass cytometry dataset in the public domain. **A** Workflow for assessing the degree of marker overlap in the FlowRepository (FR) database. A total of 44 datasets were retrieved with the PBMC tag from FR. Merging one representative panel from each dataset identified a total of 192 overlapping markers suitable for integration from a total of 808 panels. **B** Heatmap visualization on the degree of panel overlaps within the top 50 consensus markers based on their frequency in the retrieved panels (black: presence of the marker, red: absence of the marker). **C** Marker frequency ranking shows that >89% of the datasets have panel overlaps within the top 3 markers, >86% have panel overlaps within the top 5 markers while > 50% have panel overlap for all 20 markers. **D**, **E** Integration of three public mass cytometry datasets containing tumor-infiltrating leukocytes (TIL) across four different cancer histologies (red: breast, blue: glioma, green: kidney, yellow: sarcoma, gray: healthy control). Comparing pre- (**D**) and post-normalization (**E**) using CytofIn demonstrated improvements in mean expression clustering between the breast and within the glioma datasets, indicating a reduction of batch effects. See Supplementary Fig. 19 for detailed quantification of batch effect reductions between and within datasets based on the pair-wise RMSD values.

Flow Repository is one of the largest public repositories of mass cytometry data^10,35^. To test the utility of the CytofIn pipeline for public databases, we first examined the number of datasets that may have sufficient overlapping panels to use with CytofIn. We queried the Flow Repository database for any dataset tagged with the term PBMC. This retrieved a total of 44 mass cytometry datasets (Fig. 6A). After merging one representative panel from each dataset, we identified a total of 192 overlapping markers suitable for integration from a total of 808 panels, with 1 panel per file (Fig. 6A). By assessing the degree of panel overlap within the top 50 consensus markers based on their frequency in the retrieved panels, we showed that > 89% of the datasets have panels that overlap within the top 3 markers, which can be suitable for CytofIn integration (Fig. 6B).

Differences in metal-antibody pairing across cohorts following panel homogenization could be another important source of technical variation and a limitation to dataset integration. Across the 44 PBMC datasets from Flow Repository, we surveyed the frequency of metal-antibody pairing on the top five most common overlapping markers. We found that 4 of 5 were paired with the same metal over 50% of the time (CD3, CD4, CD8, CD19) (Supplementary Fig. 16). We analyzed the impact of differences in metal-antibody pairing on these four markers in two publicly available PBMC datasets from patients with melanoma. Between the two panels, these four antibodies were present in both panels but between the two panels, only one marker was labeled on the same metal (CD19). We quantified the differences in metal sensitivity as previously reported (Supplementary Fig. 17A)^36^. Using files from patients treated with Pembrolizumab at 3 weeks from both datasets, we evaluated the mean marker expression relative to the metal sensitivity difference between the two panels. The mean expression of these markers was not significantly different before and after batch normalization (Supplementary Fig. 17B). Biaxial plots demonstrate the preservation of these populations after normalization with CytofIn (Supplementary Fig. 17C).

To determine CytofIn’s utility in datasets collected across tissue types, we applied CytofIn to three datasets analyzing tumor-infiltrating leukocytes (TIL) across four different cancer histologies (breast^37^ and Flow Repository ID FR-FCM-ZYJP; and glioma, kidney, sarcoma^38^). The datasets shared 11 consensus markers and CytofIn used the top three stable channels for normalization. We found that before normalization, the TIL’s from the breast cancer samples did not cluster together but following CytofIn normalization these samples clustered together and away from glioma, a known cold tumor with fewer infiltrating immune cells (Fig. 6D, E). Similarly, improvements in clustering were also observed within the breast and glioma datasets. Quantification of the clustering quality showed that the adjusted Rand Index increases from 0.48 to 0.61 post CytofIn normalization by comparing to designated cancer type labels. Likewise, pairwise RMSD values of the mean expression profile between samples for each cancer type consistently decreased post CytofIn normalization, indicating a decrease in batch variation (Supplementary Fig. 18). This combined analysis of infiltrating immune cells from patients of multiple cancer types demonstrates the feasibility of CytofIn across tissue types from different studies.

### CytofIn dataset integration uncovers immunotherapy correlates

With the growing use of mass cytometry for immune monitoring in the context of immunotherapy treatment for cancer, we analyzed two datasets from Flow Repository examining the effects of immune checkpoint inhibition on peripheral blood immune populations in melanoma patients^39,40^. Greenplate et al. demonstrated that treatment with the PD-1 inhibitor Pembrolizumab results in a reduction of CD4^+^PD1^+^ T cells and CD8^+^PD1^+^ T cells compared to the pretreatment samples^39^. Conversely, Wei et al. found CD8^+^PD1^+^ T cells were expanded in PBMC samples of melanoma patients treated with Nivolumab, Pembrolizumab, or Ipilimumab (a CTLA4 inhibitor) monotherapy, but the effects of these immunotherapies on CD4^+^PD1^+^ T cells were not well-characterized^40^. To investigate how each immunotherapy affects T cells and other immune populations in melanoma patients, we integrated these datasets for combined analyses: dataset 1 containing the pretreatment and Pembrolizumab-treated PBMC’s from Greenplate et al. and dataset 2 containing Pembrolizumab, Nivolumab, Ipilimumab, or a combination of Ipilimumab and Nivolumab treated PBMC samples by Wei et al. (Supplementary Data 3).

**Fig. 7:**
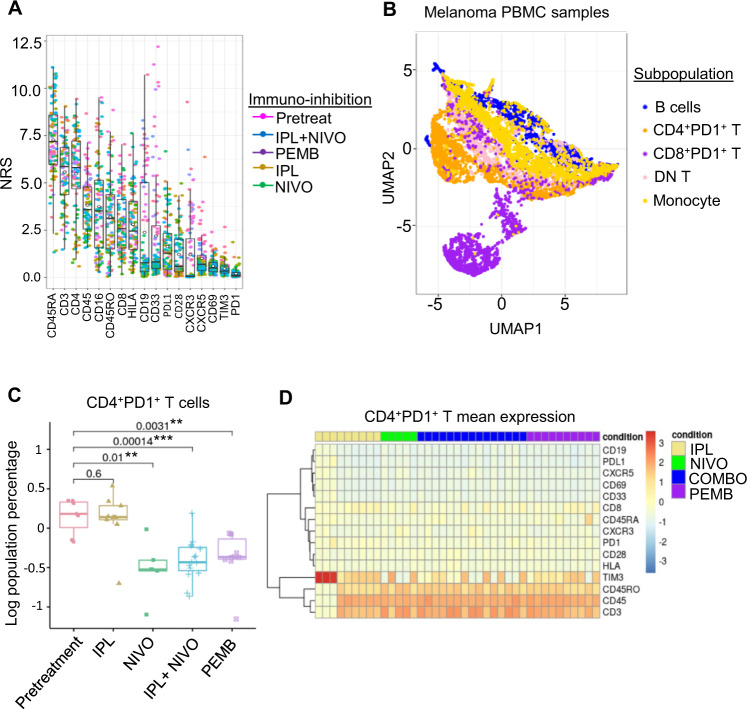
Integration of melanoma datasets to uncover cellular correlates of immunotherapy. **A** Channel stability determination using a PCA-based non-redundancy score to rank order marker variability among melanoma PBMC samples under immunotherapy from two independent melanoma datasets (*n* = 74). The band indicates the median, the black circle indicates the mean, the box indicates the first and third quartiles and the whiskers indicate ± 1.5 × interquartile range. Dataset 1 contains pretreatment and Pembrolizumab (PMB)-treated PBMCs and dataset 2 contains Pembrolizumab (PMB), Nivolumab (NIVO), Ipilimumab (IPL), or a combination of IPL and NIVO-treated PBMC samples. **B** tSNE visualization of 8 major melanoma PBMC subpopulations based on gating strategy defined in dataset 1. **C** Comparison of CD4+PD1+ T cell abundance in pretreatment (*n* = 7) or immunotherapy treated patients with IPL (*n* = 9), NIVO (*n* = 5), IPL+NIVO (*n* = 15), or PEMB (*n* = 10) after 3 weeks of treatment using a two-sided Wilcoxon test (*p*-values: *<0.05, **<0.01, ***<0.001). The band indicates the median, the box indicates the first and third quartiles and the whiskers indicate ± 1.5 × interquartile range. **D** Expression of 15 markers within the CD4+PD1+ T cells. The colors are scaled based on the expression ratio of treatment to pretreatment samples.

To analyze these datasets together, the FCS files from each dataset were homogenized to a panel of 17 consensus markers. To minimize batch effects, MSFTB batch normalization was performed using the top three stable channels: PD1, TIM3, and CD68 as generalized anchors ranked by NRS (Fig. 7A). To define each T cell population, we gated samples from both datasets into eight immune subpopulations using the gating strategy outlined by Greenplate et al. (Fig. 7B)^39^. To ensure that the signals between the two datasets were comparable post batch normalization, we first compared the population frequency of pembrolizumab-treated samples at the week 3 time point from both datasets. We saw an increase in the correlation between T cell subpopulation frequencies from the two datasets after performing our batch normalization procedure (R^2^ values increase from 0.89 to 0.92) (Supplementary Fig. 19A). Similarly, the difference in the T cell subpopulation frequency between the corresponding samples decreased after batch normalization (average RMSD values decrease from 0.29 to 0.27) (Supplementary Fig. 19B). Importantly, the frequency of T cells, particularly CD4^+^ T cells, were most impacted by the batch normalization and is the subpopulation that exhibited the highest sensitivity to immunotherapy from both studies (Supplementary Fig. 19B).

Our integrative analysis showed that Nivolumab monotherapy or combination therapy with Ipilimumab resulted in a significant reduction in the CD4^+^PD1^+^ T cells similar to the effect of Pembrolizumab as previously observed in Greenplate et al. (Fig. 7C). There were no significant changes in CD4^+^PD1^+^ T cell frequency in Ipilimumab-treated patients when compared to their pretreatment sample (Fig. 7C). Although all anti-PD1 therapies appeared to reduce CD8^+^PD1^+^ T cell abundance, the changes were not as significant as in the CD4 + PD1 + T cell subpopulation (Supplementary Fig. 20). Interestingly, an expansion in the CD8 + T cell population was seen in the Nivolumab but not the Pembrolizumab-treated samples (Supplementary Fig. 20). In CD4^+^PD1^+^T cells, 11 out of 15 proteins had reduced expression after immune checkpoint inhibition compared to the pretreatment PBMC samples, including CXCR5 and CD69 (Fig. 6D and Supplementary Fig. 21). On the other hand, CD45RO, CD45, and CD3 were elevated across all treatments (Fig. 7D and Supplementary Fig. 21). Interestingly, TIM3 was elevated only in a subset of the Ipilimumab-treated samples but not in the anti-PD treatments (Fig. 7D and Supplementary Fig. 21). This integrative analysis was able to capture both immune features reported in the original study as well as on subpopulations not previously defined in the original study via combined analysis. Our approach enabled cross-study comparison to reveal single-cell features that exhibit a differential response to immunotherapy.

## Discussion

Batch normalization across mass cytometry datasets remains a major bottleneck for performing large-scale data integration from public databases. Current approaches for mass cytometry data normalization across datasets often demand the use of identical replicates or bead standards and whose absence can hamper cross-dataset comparison. To address these challenges, we presented a data integration strategy combining batch channel homogenization and data normalization using generalized anchors for comparative analysis of mass cytometry data. We validated our approach by comparing batch normalization outcomes with that by bead normalization across 989 leukemia samples and showed that our approach can accurately recapitulate the result of bead normalization, the current gold standard for mass cytometry normalization. Similarly, we showed that our approach had minimal effect on the subpopulation frequency and protein marker distribution while achieving high consistency and robustness at the single-cell level compared to the current gold standard, bead normalization. To demonstrate the utility of our approach for mining publicly available mass cytometry datasets, we implemented CytofIn to integrate three tumor-infiltrating lymphocyte datasets and two melanoma datasets from FlowRepository and identified immune features from melanoma patients sensitive to immune checkpoint inhibition.

Several approaches for batch normalization have been developed that obviated the use of bead standards. Methods like BatchAdjust compute a scaling factor between protein measurements in each sample and a shared reference sample to make sample adjustments in each batch^19^. Given that single batch adjustment based on the bulk expression may not accurately reflect the adjustment needed at the single-cell level, CytoNorm was then proposed^18^. In this approach, the batch adjustment was achieved based on a cell cluster generated by FlowSOM, which is then used to define cluster-specific goal distribution for fitting and transforming each protein measurement. Other methods like CytofRUV used landmark proteins to improve consistency between batches^28,41^. However, for cross-dataset analysis from public databases, the requirement of identical replicates in each batch is a major limitation underlying these methods. Recent deep learning approaches such as distribution-matching residual networks or multi-tasking neural networks offer potential solutions, yet these methods require substantial computation time for parameter tuning tailored for specific training data thus limiting their general use^20,42^. Methods like Seurat risk losing biological signals as they merely integrate expression data based on mutual nearest neighbor without using meaningful anchors.

Our study demonstrated the potential utility of generalized anchor-based batch normalization, implemented as CytofIn, for fast and robust integrated analysis of public mass cytometry databases. We found that several types of unexpected controls, including healthy samples and stable channels, were suitable anchors for batch normalization. These anchors exhibit small variability between batches yet remain robust control for monitoring batch variations. When tested on large cohorts of leukemia patient samples, we showed that batch effects can be systematically reduced. Since the stable channels were identified from shared markers between samples, CytofIn obviates the need for pre-selected housekeeping or landmark markers. Also, in contrast to deep learning approaches, CytofIn does not require pretraining and can be easily integrated into existing mass cytometry workflows. Additionally, we showed the batch correction can be accurately propagated to the subpopulation level even though the signal adjustment was performed on the bulk expression data.

We have proposed five batch normalization functions including MSFT, MSFTB, VAR, Z, and BL where each has different effects on marker distribution, cell abundance, mean expression as well as batch and cell mixing. In particular, MSFTB and BL normalization perform most similarly to bead normalization with the lowest RMSD values (Supplementary Fig. 9B). Although MSFTB appears to be the optimal normalization from our study, BL normalization has led to a greater reduction in variance values between samples after normalization (Supplementary Fig. 5). On the other hand, normalization based on VAR and Z deviated significantly from the bead normalization result possibly due to the strong assumption of equal variance between healthy samples. However, we anticipate that these functions could be useful when identical control samples were used, as a special case of generalized anchors. By comparing local and global label distribution, we showed that Z and MSFT normalization increase batch mixing over bead normalization while MSFT, MSFTB, and BL are the top three methods that are closest to bead normalization based on kBET and LISI index. MSFTB and Z normalization were found to be best at maintaining cell type specificity among the five normalizations. The performances of five CytofIn normalization functions against different metrics evaluated in our study are summarized in Supplementary Fig. 22.

In conclusion, CytofIn is a fast, accurate, and robust mass cytometry data integration pipeline that supports CyTOF data standardization and batch normalization without necessitating bead information or identical technical replicates. The high flexibility of our framework enables multiple forms of generalized anchors as well as normalization functions to be developed. In addition to healthy controls and stable channels considered in this study, the generalized anchor can be extended to include stable cell subpopulations identified across analyzed samples. Recent advances in embedding techniques also hint at the possibility of using more abstract anchors generated by autoencoders on an embedded space^42^. Although mean expression and variance values are robust proxies for data distribution, non-linear transformations for direct mapping of probability density between samples or using a more realistic representation, such as Gaussian mixture models may further improve the resolution of batch corrections. While our approach is currently limited to datasets with shared markers, imputation techniques like nearest neighbor clustering could be used to infer non-overlapping markers thus expanding the applicable datasets^43–45^. Identification of shared markers can be automated using natural language processing, such as computing similarity between the labels using weighted edit-distances followed by the Hungarian assignment algorithm for finding an optimal bipartite matching. While metal tag-antibody pairings may be a limitation across all shared markers, we did not find differential metal sensitivity a more prominent source of variability than other potential sources of batch effects such as antibody staining or instrument sensitivity, and we demonstrate the ability to identify comparable populations even while labeled with different metals. Regardless, researchers should evaluate populations carefully across panels to understand the impact of metal tag-antibody labels between datasets. Further, with the increasing availability of harmonized data^46^ and commercially available antibody panels, more and more datasets will have consistent metal tag-antibody pairings, easing these comparisons. Our batch normalization approach is not limited to mass cytometry data but can be potentially applicable for diverse datasets such as genomic data, RNA-seq data, flow cytometry data, and spatial mass cytometry data. Finally, with the increasing availability of publicly available data from clinical mass cytometry experiments, we expect that CytofIn will be useful to aid in large-scale CyTOF data integration and enable predictive modeling across large clinical cohorts.

## Methods

### Primary samples

De-identified bone marrow or peripheral blood primary samples from patients with ALL or lymphoma were obtained under informed consent from Lucile Packard Children’s Hospital and Stanford Hospital at Stanford University (Stanford, CA, USA) and from the Pediatric Clinic of University of Milano-Bicocca (San Gerardo Hospital, Monza, Italy) and from St. Jude Children’s Research Hospital (Memphis, TN, USA). The use of these samples was approved by the Institutional Review Boards at each institution. Cryopreserved primary bone marrow and peripheral blood samples, from leukemia or lymphoma patients or healthy controls, thawed rapidly in thawing media (RPMI 1640 supplemented with 10% fetal bovine serum, 1% penicillin-streptomycin, and glutamine, 20 U/mL sodium heparin, and 0.025 U/mL Benzonase)^6^. Cells were rested for 30 min at 37 °C and cisplatin viability stained^47^. Cells then underwent ex vivo perturbation as shown in Supplementary Data 1.

### Mass cytometry

After ex vivo perturbation, cells were fixed with paraformaldehyde, washed in cell staining media (CSM) twice, followed by one wash in PBS and one wash in PBS + 0.02% saponin. Cells were then 20-plex barcoded, using new in-house batch preparations. Aliquots of the same healthy BM controls were used, one per barcode plate. Samples were washed in CSM after barcoding and combined into a single tube. Blocking was performed with Human TruStain FcX receptor blocking solution (Biolegend, 422302). Cells underwent surface staining with surface markers outlined in Supplementary Table 2. Following surface staining, cells were washed, permeabilized, and intracellular stained (Supplementary Table 2). Once intracellular stained, samples were washed in CSM, Iridium intercalated, washed in CSM, followed by two washes in ultra-pure double-distilled water. To prepare for acquisition, cells were resuspended with normalization beads^16^. Cohorts 1–5 were acquired on a CyTOF1 generation machine, while Cohorts 6 and 7 were acquired on the Helios (3^rd^ generation CyTOF).

### Data processing

Data acquired for internal cohorts on the CyTOF1 was downloaded and re-processed along with external cohorts acquired from the Helios. Due to the limitations of the first-generation instrument, some FCS files were re-processed to include the IMD header, re-extracted from IMD and concatenated through the Fluidigm software. Once all barcoded files for internal and external cohorts were concatenated into single individual files, the data was bead normalized^16^. Following bead normalization, the FCS files were de-barcoded into individual samples^48^.

### File homogenization

Before data normalization, the antigen panels in FCS files were homogenized. Briefly, a standard antigen panel containing metal name, antigen name, regular expression pattern, and the standardized name was generated (Supplementary Table 2). The regular expression pattern was used to search and standardize antigen names. We have implemented a computational pipeline for data homogenization and batch normalization and is accessible from the CytofIn R package (https://github.com/bennyyclo/cytofin).

### CytofIn batch normalization algorithm

CytofIn implements two strategies for batch normalizing CyTOF datasets. The first of these strategies uses healthy control samples (1 per barcoding plate) as generalized anchors to batch correct each plate relative to the universal reference. The universal reference is obtained by concatenating and averaging the data from each plate’s generalized anchor. The second of CytofIn’s batch normalization strategies uses the combined datasets’ most stable (i.e., least variable) channels as generalized anchors in order to batch correct all other antigen channels in the consensus CyTOF panel. The implementation of these strategies is discussed in-depth below:

Batch normalization using healthy control samples: CytofIn batch normalization using healthy control samples as generalized anchors is performed in 3 steps. First, one control sample per batch is identified as that batch’s generalized anchor. Second, universal reference statistics are computed using the combined single-cell data from all generalized anchors. Third, the differences in marker expression means and/or variances between each generalized anchor and the universal reference are used to define a normalization function (from a choice of 5 functions) that adjusts each generalized anchor to match the universal reference. Finally, the same normalization function is then applied to all other samples of the same batch until all samples are batch normalized. The mathematical details of this procedure are outlined in the Supplementary Methods.

Determination of the universal reference. The universal reference for batch normalization is computed by concatenating the single-cell data from all control samples to form an expression matrix $X\in {R}^{{c}_{{control}}\times m}$, where *c*_*control*_ is the total number of cells across all control samples and *m* is the number of markers in the consensus antigen panel. The mean signal intensity (MSI) of the universal reference, ${MS}{I}^{({universal})}\in {R}^{m}$, can then be defined by finding the column means of X as follows:

1 $$MS{I}^{(universal)}=\frac{{\sum }_{j=1}^{{c}_{control}}{X}_{[j,1:m]}}{{c}_{control}}$$

where X_[*j*, 1*:m*]_ represents an m-dimensional vector corresponding to the *j*th row of X. Likewise, the vector of marker variances ${Va}{r}^{({universal})}\in {R}^{m}$ across all cells in the universal reference can be calculated as follows:

2 $$Va{r}^{(universal)}=\,\frac{{\sum }_{j=1}^{{c}_{control}}{({X}_{[j,1:m]}-MS{I}^{(universal)})}^{2}}{{c}_{control}}$$

Both MSI^(universal)^ and Var^(universal)^ are used in the next step of the algorithm.

Fitting healthy control samples as generalized anchors to the universal reference. We have proposed five normalization functions for fitting the mean expression of generalized anchors to the universal reference based on a combination of the anchors’ and reference’s mean and variance values.

First, we define the mean signal intensity (MSI) of the control sample from batch *j*, which we denote as ${MS}{I}_{j}^{\left({control}\right)}=\left\{{x}_{1},{x}_{2},\ldots ,{x}_{m}\right\}\in {R}^{m}$ where each element ${x}_{i}\in R$ is the mean expression of marker *i* across all cells in the generalized anchor (i.e., control sample) from batch *j*. Similarly, we define the marker variance vector across all cells in the control sample from batch *j*, which we denote as ${Va}{r}_{j}^{\left({contro}l\right)}=\left\{{v}_{1},{v}_{2},\ldots ,{v}_{m}\right\}\in {R}^{m}$ where each element ${v}_{i}\in R$ is the variance of marker *i* across all cells in the generalized anchor (i.e., control sample) from batch *j*.

For each batch *j*, we also define the single-cell expression matrix obtained by concatenating (row-wise) cells from all samples within batch *j*. We denote this expression matrix as

$${T}_{j}\in {R}^{{c}_{j}\times m}$$

where *c*_*j*_ is the total number of cells in batch *j* and m is the number of markers in the consensus panel.

Using these definitions, we can then apply any of the following batch normalization functions to the data from batch j to estimate and correct batch effects:

Meanshift normalization. This function performs per-channel additive adjustment to each entry in T_j_ based on the differences between the entries of ${MS}{I}_{j}^{({control})}$ and ${MS}{I}^{\left({universal}\right)}$. The batch-corrected expression matrix ${T}_{j}^{({corrected})}\in {R}^{{c}_{j}\times m}$ is generated as follows:

3 $${T}_{j}^{({corrected})}={T}_{j}+\left({MS}{I}_{j}^{\left({contro}l\right)}-{MS}{I}^{\left({universal}\right)}\right)$$

where the difference on the right-hand side of the equation is broadcasted to each row of T_j_.

Meanshift bulk normalization. This function performs additive adjustment based on the difference in the mean (across markers) of ${MS}{I}_{j}^{({control})}$ and the mean (across markers) of ${MS}{I}^{\left({universal}\right)}$. The batch-corrected expression matrix ${T}_{j}^{({corrected})}\in {R}^{{c}_{j}\times m}$ is generated as follows:

4 $${T}_{j}^{(corrected)}={T}_{j}+\left[\left(\frac{{\sum }_{i=1}^{m}MS{I}_{j}^{(control)}}{m}\right)-\left(\frac{{\sum }_{i=1}^{m}MS{I}^{(universal)}}{m}\right)\right]$$

Variance normalization. This function first performs additive correction (as in meanshift normalization) followed by scaling based on the ratio of the standard deviation (SD) value of the generalized anchor from batch *j*, $S{D}_{j}^{\left({control}\right)}=\sqrt{{Va}{r}_{j}^{\left({control}\right)}}\in {R}^{m}$ to that of the universal reference. Thus, the batch-corrected expression matrix ${T}_{j}^{({corrected})}\in {R}^{{c}_{j}\times m}$ is generated as follows:

5 $${T}_{j}^{({corrected})}=\left[{T}_{j}+\left({MS}{I}_{j}^{\left({control}\right)}-{MS}{I}^{\left({universal}\right)}\right)\right]* \frac{S{D}^{\left({universal}\right)}}{S{D}_{j}^{\left({control}\right)}}$$

Z-score normalization. This function performs Z-score standardization. The batch-corrected expression matrix ${T}_{j}^{({corrected})}\in {R}^{{c}_{j}\times m}$ for batch *j* is generated as follows:

6 $${T}_{j}^{({corrected})}=({T}_{j}-{MS}{I}_{j}^{({control})})* \frac{S{D}^{({universal})}}{S{D}_{j}^{({control})}}+{{MSI}}^{({universal})}$$

Bead-like normalization. This function performs a multiplicative correction based on the slope between ${MS}{I}_{j}^{({corrected})}$ and ${{MSI}}^{(universal)}$_._ The slope was approximated by regression analysis. The batch-corrected expression matrix ${T}_{j}^{({corrected})}\in {R}^{{c}_{j}\times {m}}$ for batch *j* is generated as follows

7 $${T}_{j}^{({corrected})}={T}_{j}* {Reg}\left(\frac{{MS}{I}^{({universal})}}{{MS}{I}_{j}^{({control})}}\right)$$

where ${Reg}$ denotes the regression function.

Batch normalization using stable channels. CytofIn batch normalization using stable channels as generalized anchors is performed in 3 steps. First, the most stable channels across all datasets being integrated are identified using a principal components-based non-redundancy score (NRS)^33,34^. Second, the n most stable channels in the combined dataset are used to establish a universal reference that can be used to batch correct the expression values of all samples in the combined dataset. Finally, the meanshift bulk normalization function is used to perform the batch correction for all samples in the combined dataset. Each of these steps is discussed in more detail below:

Identification of stable channels to be used as generalized anchors. To identify the most stable channels across multiple samples from different batches, we first express each sample s as a single-cell expression matrix, ${T}^{(s)}\in {R}^{{c}^{(s)}\times m}$, where *c*^*(s)*^ is the number of cells in sample s and m is the number of markers in the consensus antigen panel. For each sample, the top three principal components (PCs) of the expression matrix **T**^**(s)**^ are then computed to yield the top three PC loadings for each marker. Doing so results in the matrix ${P}^{(s)}\in {R}^{m\times k}$ of principal component loadings on each antigen, where m is the number of markers in the consensus antigen panel and *k* is the number of principal components (for our analyses, generally *k* = 3). In addition, the standard deviation (SD) vector $S{D}^{(s)}\in {R}^{k}$ for each PCA loading is also computed. Using these quantities, the variability of each marker *j* can then be rank-ordered using a PCA-based non-redundancy score (NRS) as previously described^34^:

8 $${{{{{{\rm{NRS}}}}}}}_{{{{{{\rm{j}}}}}}}=\frac{{\sum }_{s=1}^{R\,}\,{\sum }_{i=1}^{k}\,{(S{D}_{i}^{(s)})}^{2}{P}_{[j,\,i]}^{(s)}}{R}$$

where $S{D}_{i}^{\left(s\right)}$ is the standard deviation of the *i*-th PC in sample s, ${P}_{[j,i]}^{(s)}$ is the entry in the *j*-th row and *i*-th column of P^(s)^ (that is, the loading of the *i*-th PC on marker *j* in sample (s) and *R* is the total number of input samples to be integrated). Thus, each NRS_*j*_ represents the average (across all samples) of the following quantity for a given marker *j*, ${\sum }_{i=1}^{k}{(S{D}_{i}^{(s)})}^{2}\,{P}_{[j,\,i]}^{(s)}$.

Determination of the universal reference. Using the NRS_*j*_ calculations from the previous step, the universal reference can then be computed. To do so, we use the average expression of the n markers with the lowest NRS_*j*_ in the matrix T, which we obtain by concatenating all R samples’ expression matrices T^(s)^ row-wise such that $T\in {R}^{c\times m},$ where *c* is the total number of cells in the combined dataset and m is the number of markers in the consensus antigen panel. Thus, if we let *q* index the *n* most stable markers (from 1 to *n*) as defined above, we can calculate the *q*-th entry in the universal mean signal intensity vector ${MS}{I}^{\left({universal}\right)}=({MS}{I}_{1}^{({universal})},{MS}{I}_{2}^{({universal})},\ldots {MS}{I}_{n}^{({universal})})\in {R}^{n}$ as follows:

9 $$MS{I}_{q}^{(universal)}=\frac{{\sum }_{i=1}^{c}{T}_{[i,q]}}{c}$$

Likewise, we can define the *q*-th entry in each sample’s mean signal intensity vector ${MS}{I}^{\left({sample}\right)}=({MS}{I}_{1}^{({sample})},{MS}{I}_{2}^{({sample})},\ldots {MS}{I}_{n}^{({sample})})\in {R}^{n}$ as follows:

10 $$MS{I}_{q}^{(sample)}=\frac{{\sum }_{i=1}^{{c}^{(s)}}{T}_{[i,\,q]}^{(s)}}{{c}^{(s)}}$$

Finally, we can use these values of MSI^(universal)^ and MSI^(sample)^ to apply the meanshift bulk normalization function defined above and yield the batch-corrected expression matrix for sample *s*, which we denote as ${T}_{s}^{({corrected})}.$ Starting with the uncorrected single-cell expression matrix for batch *s*, T_*s*_, we can calculate ${T}_{s}^{({corrected})}$ as follow:

11 $${T}_{s}^{(corrected)}={T}_{s}+\left[\left(\frac{{\sum }_{i=1}^{n}MS{I}_{i}^{(sample)}}{n}\right)-\left(\frac{{\sum }_{i=1}^{n}MS{I}_{i}^{(universal)}}{n}\right)\right]$$

### Computational gating of subpopulations

Prior to computational gating, the raw mass cytometry data were transformed using the arsinh function with a scaling factor of 5. The gating parameters were optimized on the healthy samples: bone marrow mononuclear cells for B-cell leukemia or the pretreatment PBMC sample for melanoma. Each subpopulation was gated based on the biaxial plot as previously reported^6,39^. As a proxy to manual gating for the developmental classification of ALL samples, we applied rectangular gates and a threshold of 10 counts for defining positive and negative cells. To enable controlled comparison, the same gating parameters were then applied to all samples in batch to retrieve the corresponding subpopulations. The computational gating was performed using the FlowCore and openCyto R software package.

### Benchmarking

The performance of CytofIn normalization on a combined sample consisting of three representative healthy control samples from 7 cohorts was compared to three existing normalization methods, CytoNorm, CytofRUV and Seurat^18,28,49^. 2000 cells were sampled from three healthy samples of each 7 cohorts and combined to form cohort-specific healthy samples. The original and bead normalized healthy samples were also included as negative and positive controls. Furthermore, comparisons between normalized data using generalized anchors based on healthy control, stable channels, or the combination were also performed. To evaluate local batch and cell mixing effects, 10% of the samples (200 cells) were randomly sampled and mapped onto the UMAP space defined by all 36 channels in the original healthy sample space. To assessed cell mixing, the healthy cohort samples were computationally clustered into 100 cell types using flowSOM. We compared local label distribution to global distribution using the k-nearest neighbor batch effect test (kBET) and the local inverse Simpson’s index (LISI). kBET computed local batch label distribution based on the k-nearest neighbors around each data point after SVD dimension reduction. We used the expected rejection rates (0–1) as the kBET index to quantify if the null hypothesis that all batches being equally mixed is rejected. Similar to kBET, LISI assessed local label distribution of a fixed number of k-nearest neighbors and perplexity. We used the Simpson’s index outputted from the LISI program as LISI index to quantify the diversity of label distribution in a local neighborhood.

### Statistical analysis

Statistical analysis was performed using the R statistical software (www.r-project.org). Statistical parameters for protein marker distribution, change in expression and population abundance were quantified based on their mean and standard deviation (mean ± s.d.). Comparison between mean signal intensity or population abundance of mass cytometry files was performed using the Wilcoxon test. Comparison between bead and batch normalized signal was quantified using correlation analysis quantified by the R-square values or root-mean-square deviation (RMSD). Clustering quality assessments were quantified using the adjusted rand index to compare the similarity of clustering pre- and post-normalization to the pre-assigned cancer type label based on the mean expression values.

### Software

The mass cytometry data were extracted and processed using the Fludigm CyTOF software (version 7.0). The mass cytometry data were analyzed using the following packages from the R bioconductor software (version 2.2): flowcore, ggcyto, openCyto and flowWorkspace. Network and statistical analysis were performed using the Cytoscape software (version 3.8.0) and the R statistical software (version 3.6.3). Benchmark studies were conducted using the CytoNorm, CytoRUV and Seurat (version 4.0.2) software packages and kBET, LISI and FlowSOM R packages. Data integration and normalization were performed using the CytofIn R package (https://github.com/bennyyclo/Cytofin/).

### Reporting summary

Further information on research design is available in the Nature Research Reporting Summary linked to this article.

## Supplementary information

Supplementary Information

Description of Additional Supplementary Files

Supplementary Data 1

Supplementary Data 2

Supplementary Data 3

Reporting Summary

## Data Availability

The datasets for integrative comparative analysis were retrieved from the repositories, “CyTOF mass cytometry of human glioma, kidney cancer, sarcoma, PBMC” (FlowRepository ID: FR-FCM-Z3HK),” Single-cell map of diverse immune phenotypes in the breast tumor microenvironment” (FlowRepository ID:FR-FCM-ZYJP),”Mass Cytometry of Peripheral Blood from Melanoma Patients Receiving anti-PD-1” (FlowRepository ID: FR-FCM-ZYDG) and “On-therapy PBMC samples” (FlowRepository ID: FR-FCM-ZYQR) and can be accessed from the FlowRepository database (https://flowrepository.org/)^50^. Source data underlying Figs. 2–5 i.e., the healthy control samples, raw leukemia patient samples, and lymphoma patient samples used for the CytofIn validation study are available at Zendo [10.5281/zenodo.5911417].

## Code availability

The CytofIn R package is freely accessible at Github [https://github.com/bennyyclo/Cytofin].
