# Supplementary material for: CytofIn enables integrated analysis of public mass cytometry datasets using generalized anchors
Source: Nat Commun. 2022 Feb 17;13:934. doi: 10.1038/s41467-022-28484-5 (PMC8854441; doi:10.1038/s41467-022-28484-5)
Supplement: Supplementary file 1 — Supplementary Information [file 41467_2022_28484_MOESM1_ESM.pdf]

CytofIn enables integrated analysis of public mass cytometry datasets using generalized anchors

Lo *et al.*

Supplementary Figures

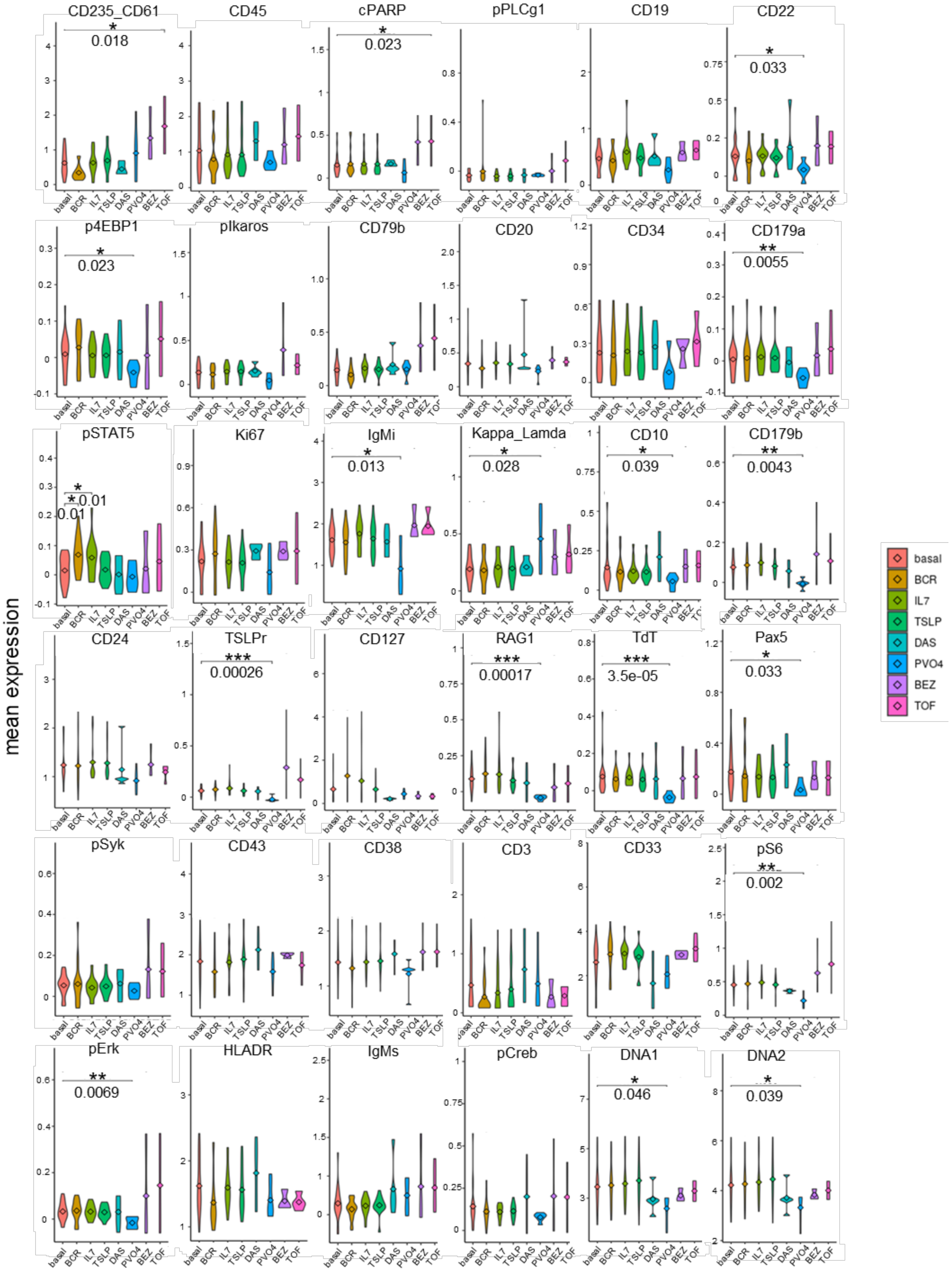

Supplementary Figure 1. Effect of *in vitro* stimulation on the protein expression of healthy control samples. To assess the effects of different stimulants on the protein expression in healthy control samples, the mean signal intensities of 36 markers in 50 healthy samples under 6 stimulation conditions (BCR, DAS, TSLP, BEZ, PVO4, TOF, and IL7) are compared to the basal condition using a two-sided Wilcoxon test (p-values: *<0.05, **<0.01, ***<0.001). The perturbation conditions include B-cell receptor cross-linking (BCR), Dasatinib (DAS), thymic stromal lymphopoietin (TSLP), BEZ-235 (BEZ), sodium orthovanadate (PVO4), tofacitinib (TOF), and IL-7 (IL7).

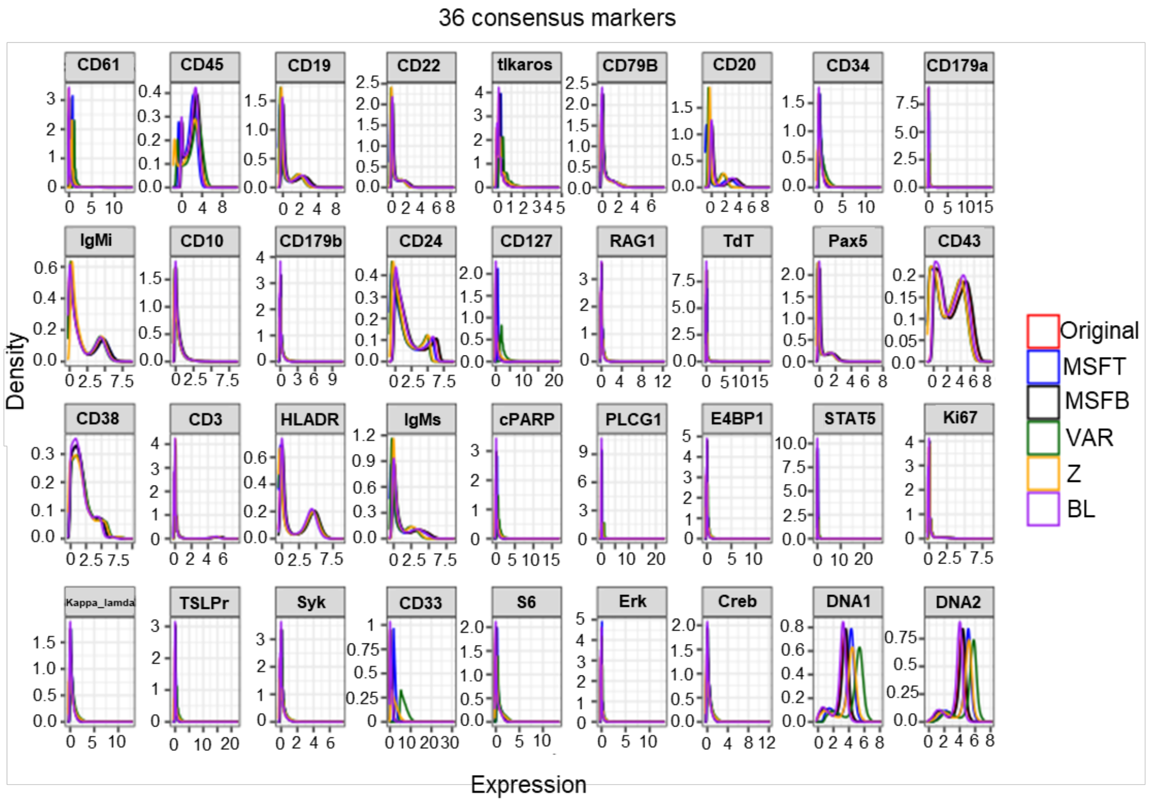

Supplementary Figure 2. One-dimensional density plots of 36 consensus markers in the healthy samples pre and post-batch normalization. Data distribution of 36 consensus markers from the aggregated healthy control samples in the basal condition (n=3). Each plot represents one protein marker comparing pre-normalized data distribution (original) to batch normalized data distribution using each of the five normalization functions: meanshift (MSFT), meanshift bulk (MSFTB), variance (VAR), z-score (Z), and beadlike (BL) normalizations.

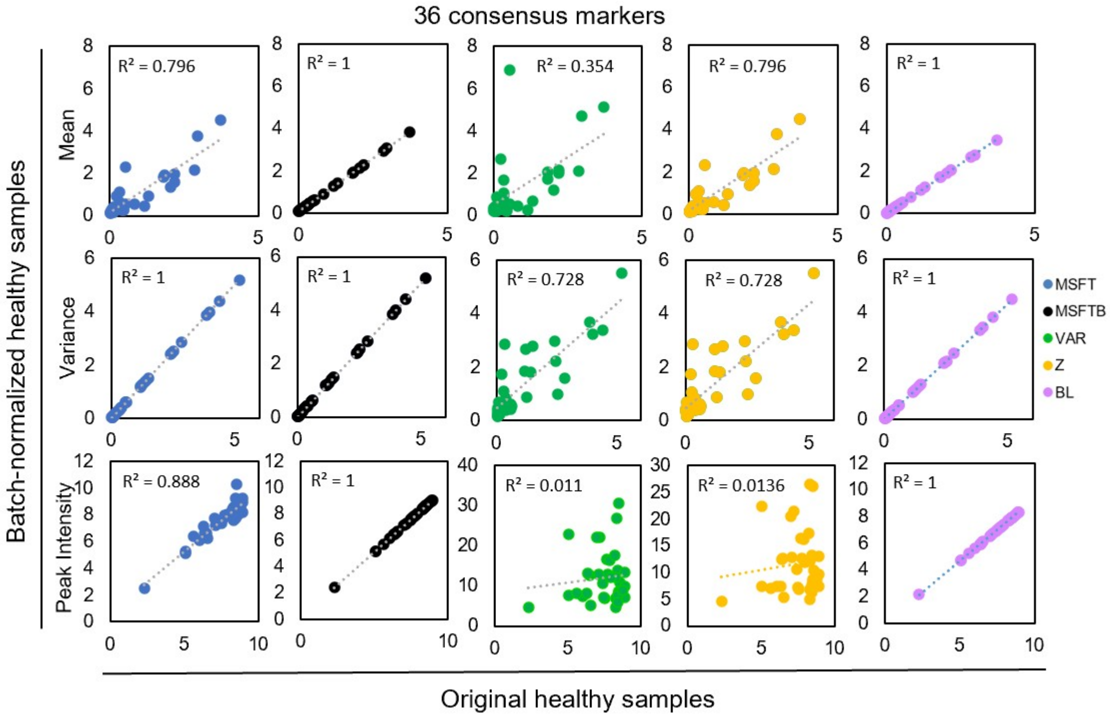

Supplementary Figure 3. Correlation analysis between original and batch-normalized data distribution of 36 markers in the healthy samples. The correlation between mean expression (row 1), variance values (row 2), and peak intensity (row 3) of 36 consensus markers pre and post-batch normalization was quantified based on the 1-d density plot (see **Figure 3C** and **Supplementary Figure 3**). For each parameter (mean expression, variance values, or peak intensity), the R^2 values were reported for each of the five normalization functions (from left to right): meanshift (MSFT, blue), meanshift bulk (MSFTB, black), variance (VAR, green), z-score (Z, yellow), and beadlike (BL, purple).

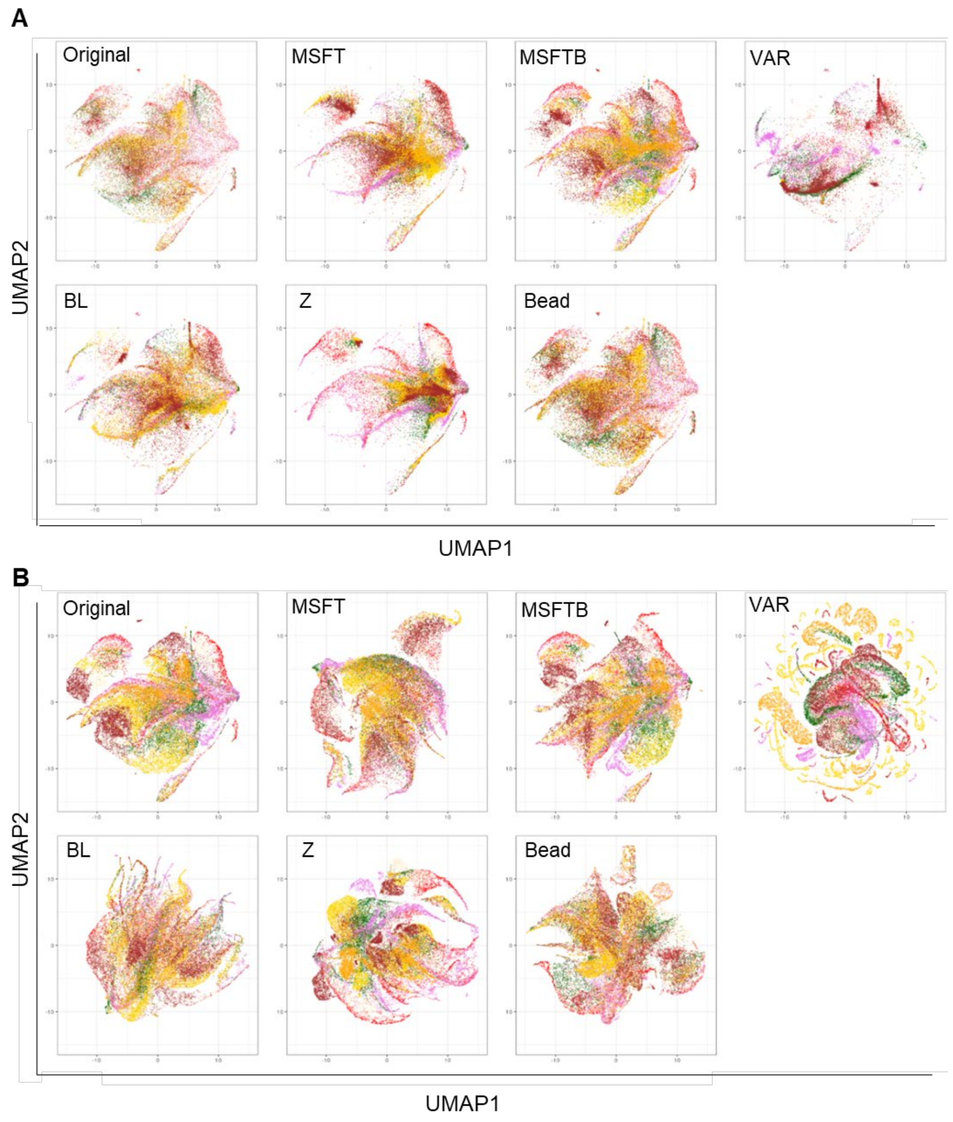

Supplementary Figure 4. Visualization of batch reduction pre and post CytofIn normalization of healthy samples from 7 cohorts. To assess the degree of batch effect reduction by each normalization function, including meanshift (MSFT), meanshift bulk (MSFTB), beadlike (BL), variance (VAR) and z-score (Z), we sub-sampled 2000 cells from three representative healthy control samples per cohort among 7 cohorts. Their single-cell distribution pre and post-batch normalization were compared using Uniform Manifold Approximation and Projection (UMAP) by projecting onto the same embedded space of the original pre-normalized samples (A) or independently (B).

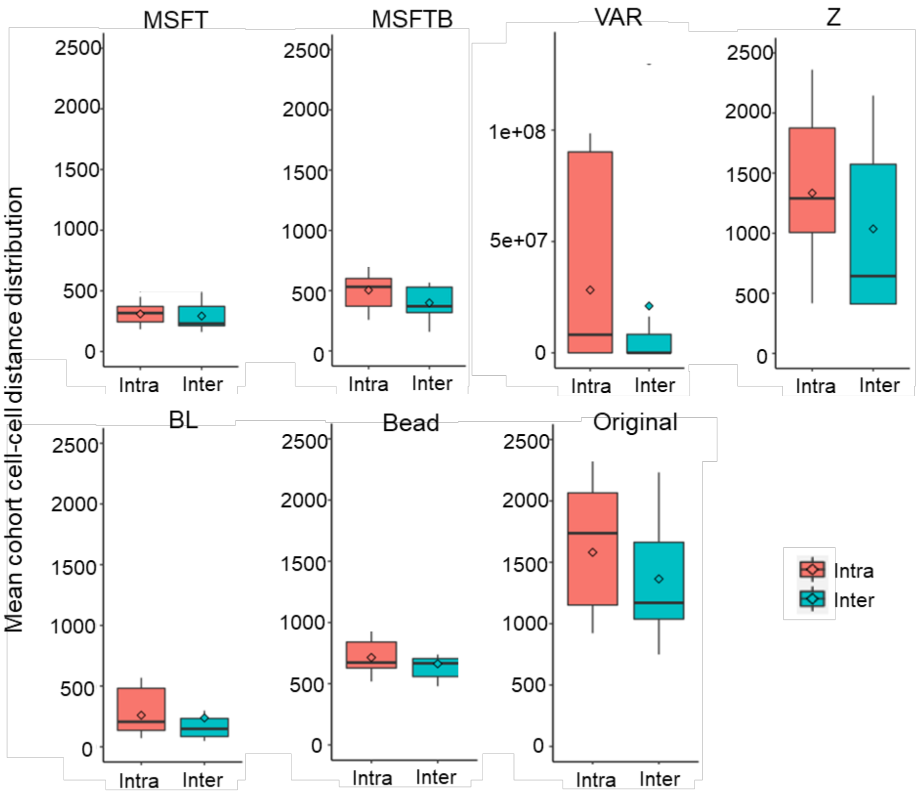

Supplementary Figure 5. Changes in cohort distance of healthy samples pre- and postbatch normalization. The difference in the mean cell-cell pairwise Euclidean distance was measured within and between 7 cohorts on the mean expression to quantify the degree of batch effect reduction using each of the five normalization functions (meanshift [MSFT], meanshift bulk [MSFTB], variance, [VAR], z-score [Z], and beadlike [BL]) as well as bead normalization (bead) compared to the original, non-batch normalized data. The band indicates the median, the black cicle indicates the mean, the box indicates the first and third quartiles and the whiskers indicate ± 1.5 × interquartile range. Note that the intra- and inter-cohort distances as well as their differences were reduced after CytofIn batch normalization by MSFT, MSFTB, and BL normalizations; importantly, the reduction in both intercohort distance and intracohort distance using these transformation functions was similar to that observed with bead normalization.

A

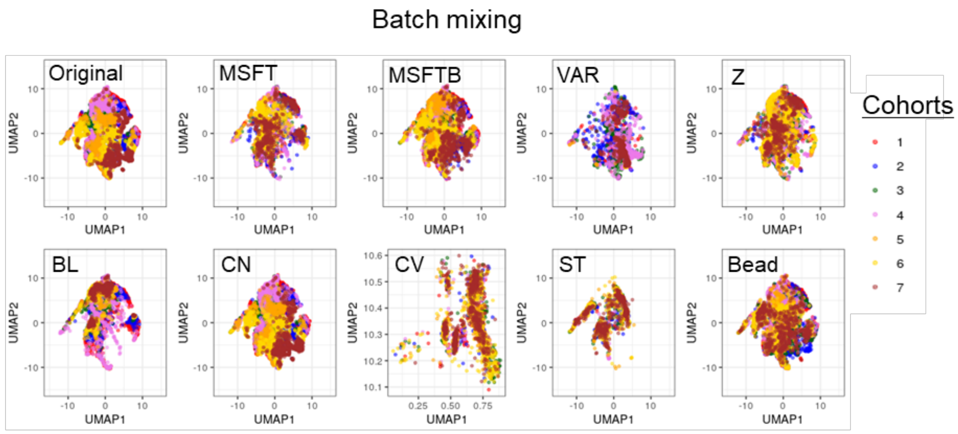

B

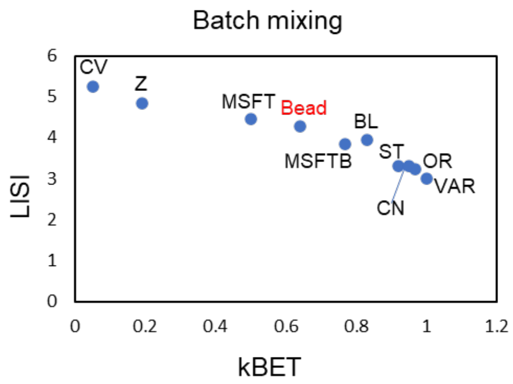

C

Batch	ΔkBET	ΔLISI
Original	+0.33	-1.05
MSFT	-0.14	+0.18
MSFTB	+0.13	-0.45
VAR	+0.36	-1.3
Z	-0.45	+0.55
BL	+0.19	-0.35
CN	+0.28	-0.97
CV	-0.59	+0.96
ST	+0.31	-0.97

Supplementary Figure 6. Benchmark study comparing batch mixing of batch normalization methods. (A) Degree of batch mixing after batch normalization of a combined sample consisting of three healthy controls from 7 cohorts using CytofIn normalization under five normalization functions: meanshift [MSFT], meanshift bulk [MSFTB], variance [VAR], zscore [Z] and beadlike [BL] compared to existing batch normalization approaches: CytoNorm [CN], CytoRUV [CV] and Seurat [ST]. (B) kBET and LISI metrics were used to quantify the degree of batch mixing by comparing the local-to-global label distribution. (C) Quantitation of batch mixing effects based on the changes in kBET and LISI index from the bead normalization.

A

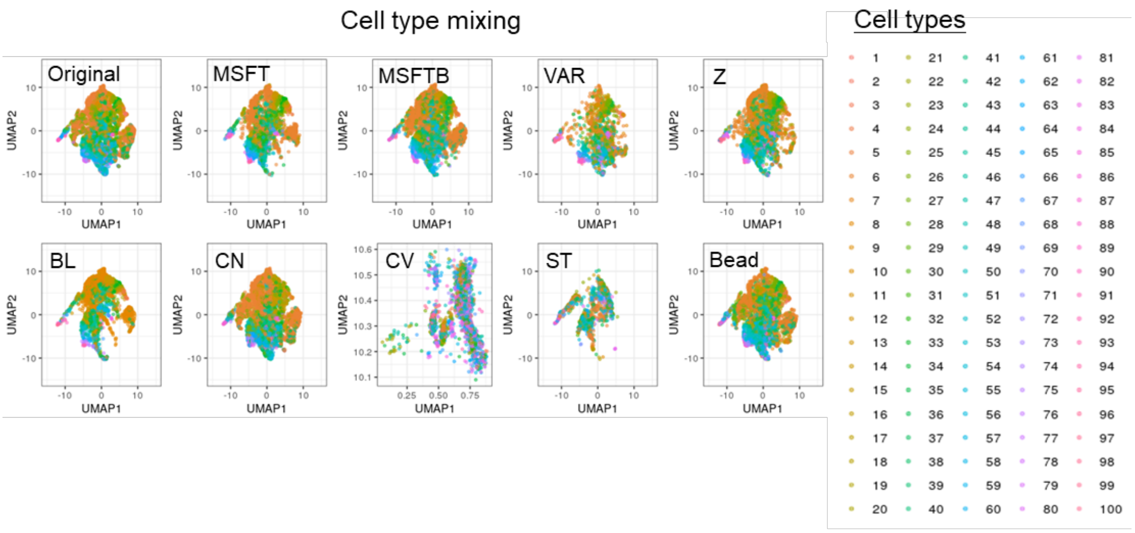

B

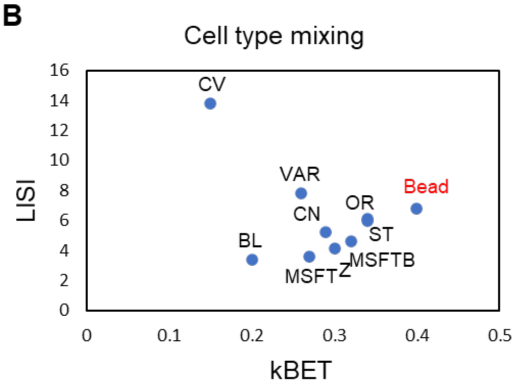

C

Cell type	ΔkBET	ΔLISI
Original	-0.06	-0.7
MSFT	-0.13	-3.21
MSFTB	-0.08	-2.18
VAR	-0.14	+1.02
Z	-0.1	-2.67
BL	-0.2	-3.41
CN	-0.11	-1.57
CV	-0.25	+7
ST	-0.06	-0.8

Supplementary Figure 7. Benchmark study comparing cell type mixing of batch normalization methods. (A) Degree of cell type mixing after batch normalization of a combined sample consisting of three healthy controls from 7 cohorts using CytofIn normalization under five normalization functions: meanshift [MSFT], meanshift bulk [MSFTB], variance [VAR], z-score [Z] and beadlike [BL] compared to existing batch normalization approaches: CytoNorm [CN], CytoRUV [CV] and Seurat [ST]. To evaluate cell type mixing of each normalization method, the combined samples were pre-clustered into 100 cell subtypes using the FlowSOM algorithm. (B) kBET and LISI metrics were used to quantify the degree of

cell-type mixing by comparing the local-to-global label distribution. (C) Quantitation of cell type mixing effects based on the changes in kBET and LISI index from the bead normalization.

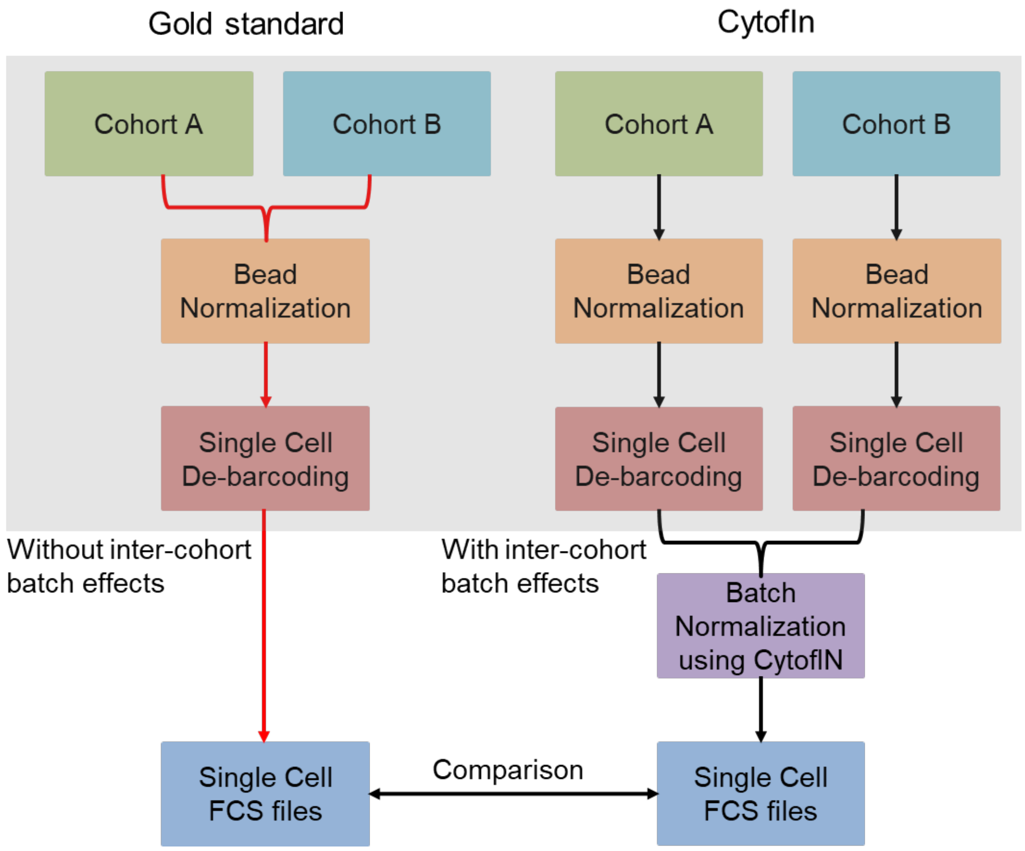

Supplementary Figure 8. Workflow for performance comparison between batch and bead normalization. To test if batch normalization can recapitulate the bead normalization performance, the 7 leukemia cohorts (cohort 1-7) consisting of a total of 989 primary patient samples were divided into two datasets: dataset A (cohort 1-4) and dataset B (cohorts 5-7). Bead normalization was performed on the two datasets together prior to debarcoding (left) or each dataset was independently bead-normalized and then normalized together by the batch normalization approach (right). Finally, normalized FCS files by bead or CyofIn batch normalization were then compared.

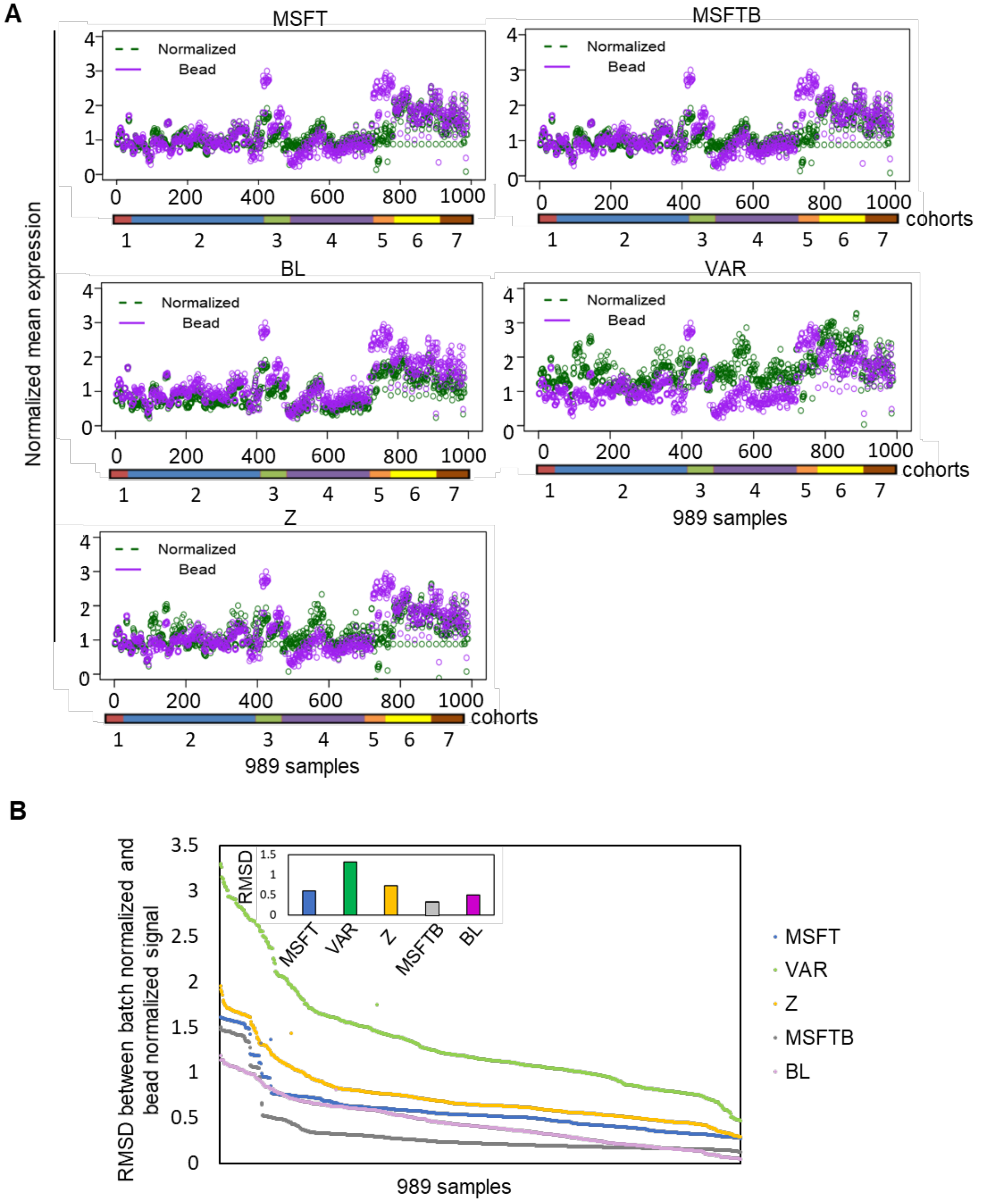

Supplementary Figure 9. Performance comparison between batch and bead normalization of 989 leukemia samples. (A) Average mean expression of 36 consensus markers from 989

leukemia samples normalized by each of the five normalization functions (green) or bead standardization (purple). Note that each dot represents the average mean marker expression per patient. (B) RMSD values between bead and batch-normalized signal under each of the five normalization functions (meanshift [MSFT, blue], meanshift bulk [MSFTB, black], variance [VAR, green], z-score [Z, yellow], and beadlike[BL, purple]) and bead standardized signal. The average RMSD values across 989 samples for MSFT, MSFTB, and BL normalizations were 0.6, 0.33, and 0.5 respectively. Increased deviations were observed for VAR and Z normalizations with RMSD values of 1.32 and 0.73 respectively.

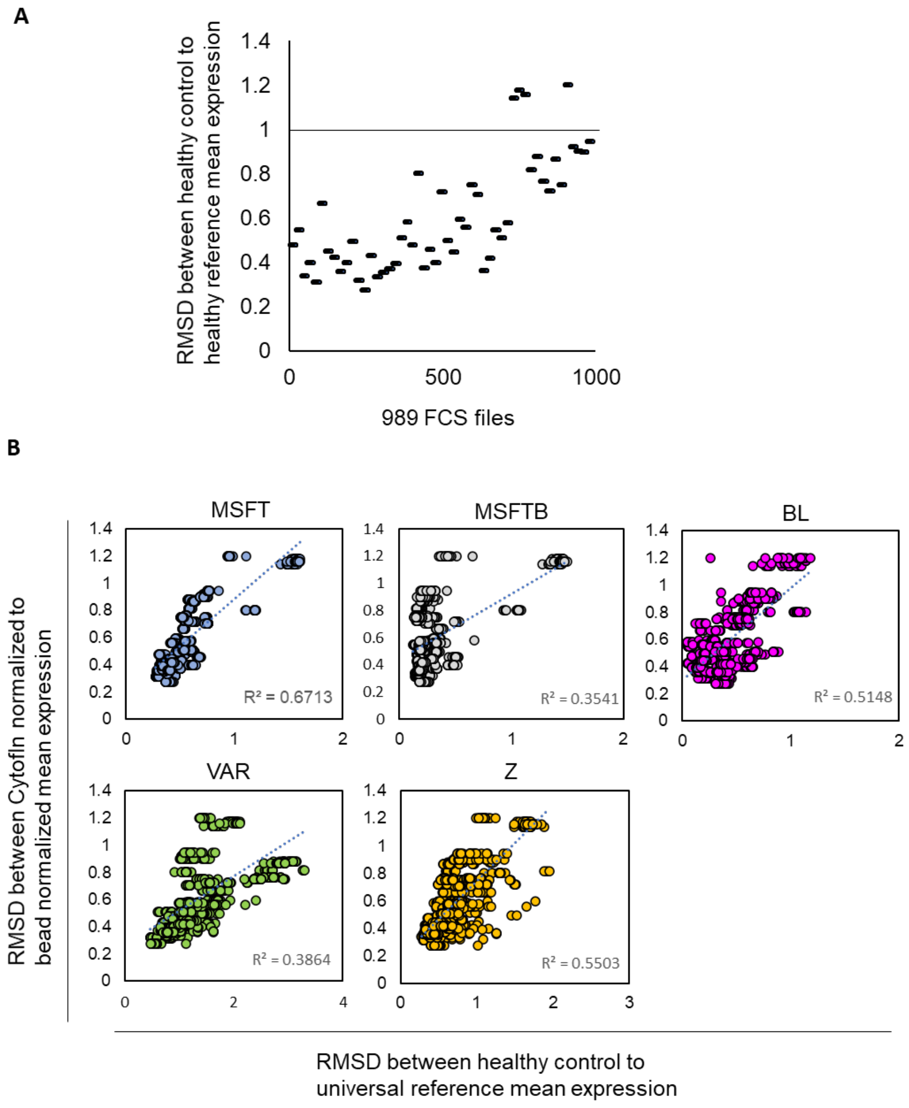

Supplementary Figure 10. Effects of healthy control anchor variability on the normalization performance. (A) The distribution of RMSD values between healthy control and universal reference mean expression across 989 primary patient samples. (B) Correlation between RMSD values of CytofIn normalized to bead normalized mean expression and RMSD values between healthy control to the universal reference mean expression for 989 primary patient samples normalized by each of the five normalization functions. Note that the performance of CytofIn normalization measured by the RMSD values is positively correlated to the variability of the healthy control samples from the universal reference.

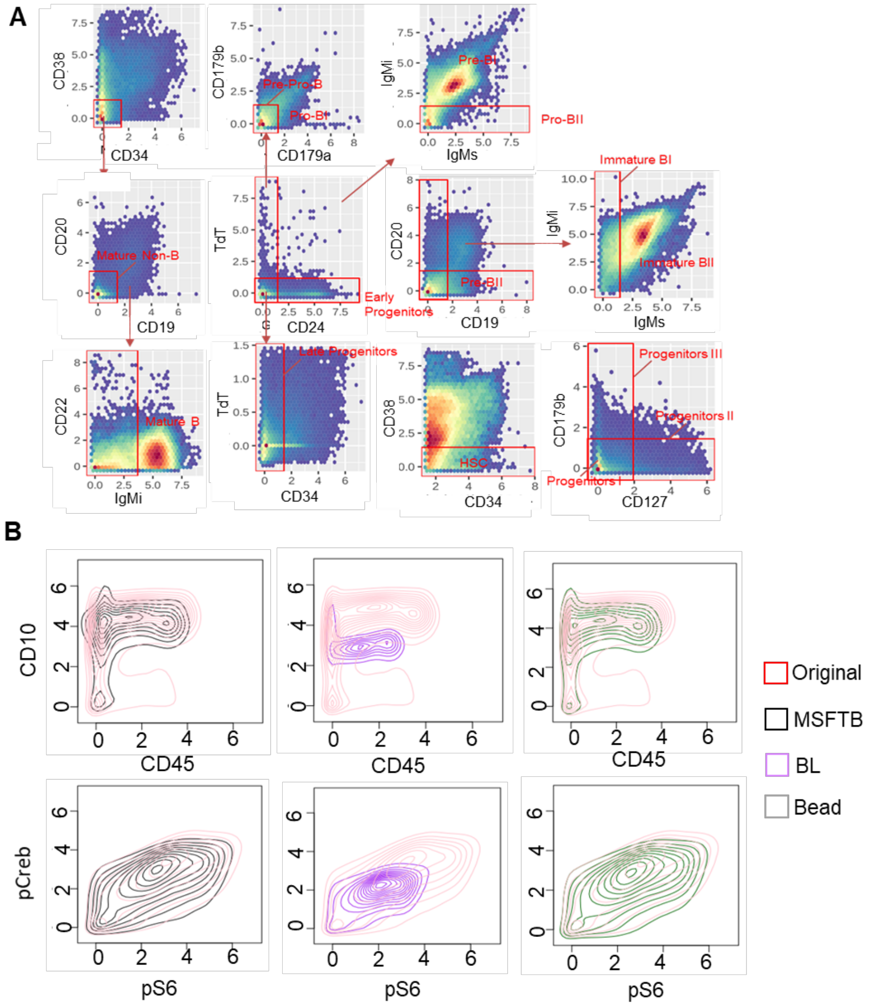

Supplementary Figure 11. Single-cell characterization of batch-normalized leukemia samples. (A) Gating strategy for 15 B-cell subpopulations. To enable controlled comparison between pre and post-normalized subpopulations, the gating parameters for each subpopulation were optimized for the healthy samples based on the biaxial plots. The same gating parameters were then applied to classify all patient samples pre and most batch normalization. (B) Comparison of biaxial plots, CD45-CD10 and pS6-pCreb markers batch normalized by meanshift bulk (MSFTB, black) and beadlike (BL, purple) normalizations and bead standardization (green) to the original (red) single-cell distribution.

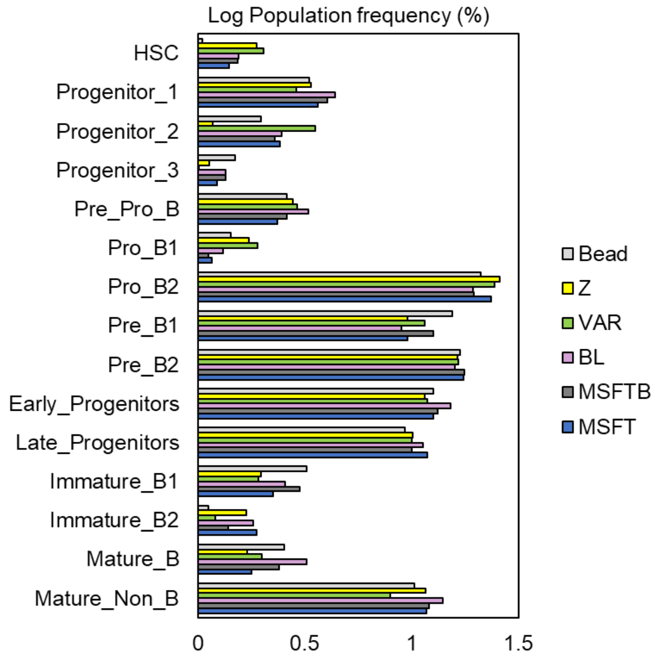

Supplementary Figure 12. Effects of batch normalization on the frequency of classified leukemia cell population. Comparison of 15 classified leukemia subpopulation frequencies batch normalized by each of the five normalization functions: meanshift (MSFT, blue), meanshift bulk (MSFTB, black), beadlike (BL, purple), variance (VAR, green), and z-score (Z, yellow) normalizations to bead normalization (Bead, gray).

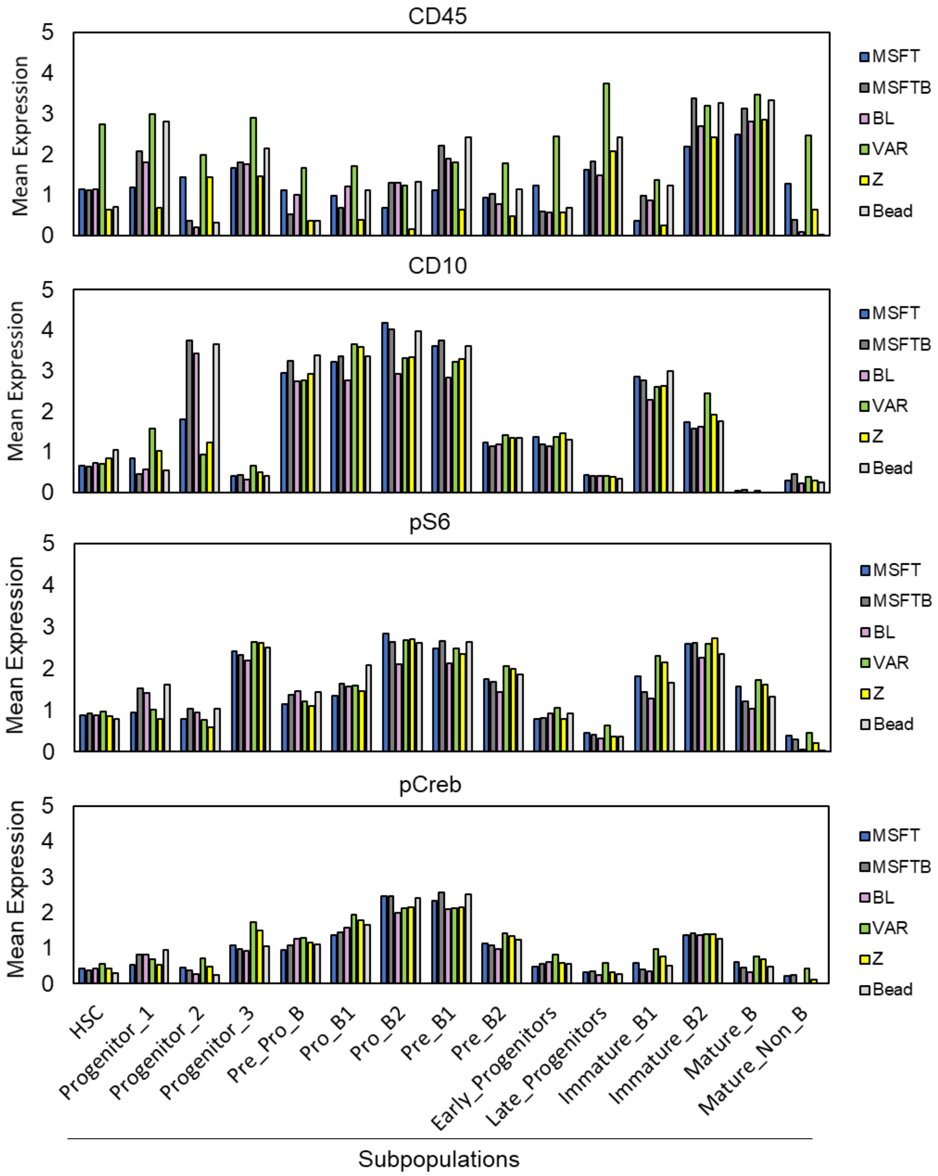

Supplementary Figure 13. Effects of batch normalization on the expression of lineage and signaling proteins in the classified leukemia cell populations. Profiling the mean signal intensity of four protein markers, including two lineage protein: CD10, CD45, and two signaling proteins: pS6, pCreb post-normalization using each of the five normalization functions: meanshift (MSFT, blue), meanshift bulk (MSFTB, black), beadlike (BL, purple), variance (VAR, green), z-score (Z, yellow) and bead standardization (Bead, gray) across 15 classified B-cell subpopulations.

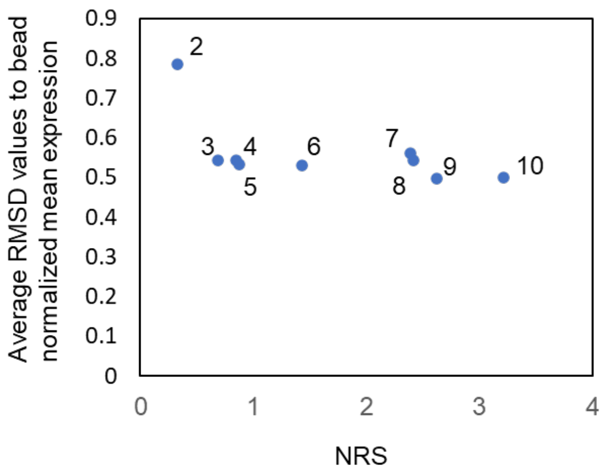

Supplementary Figure 14. Evaluation of optimal NRS cutoffs for batch normalization using stable channels. To determine the optimal NRS cutoffs for selecting stable channels for batch normalization, RMSD values were computed between the batch and bead-normalized signals by varying the NRS cutoff values. The number on each point indicated the total number of included stable channels used in CytofIn normalization. The figure shows that the average RMSD value slowly decreases with increasing NRS > 1 after the first 3-4 included stable channels.

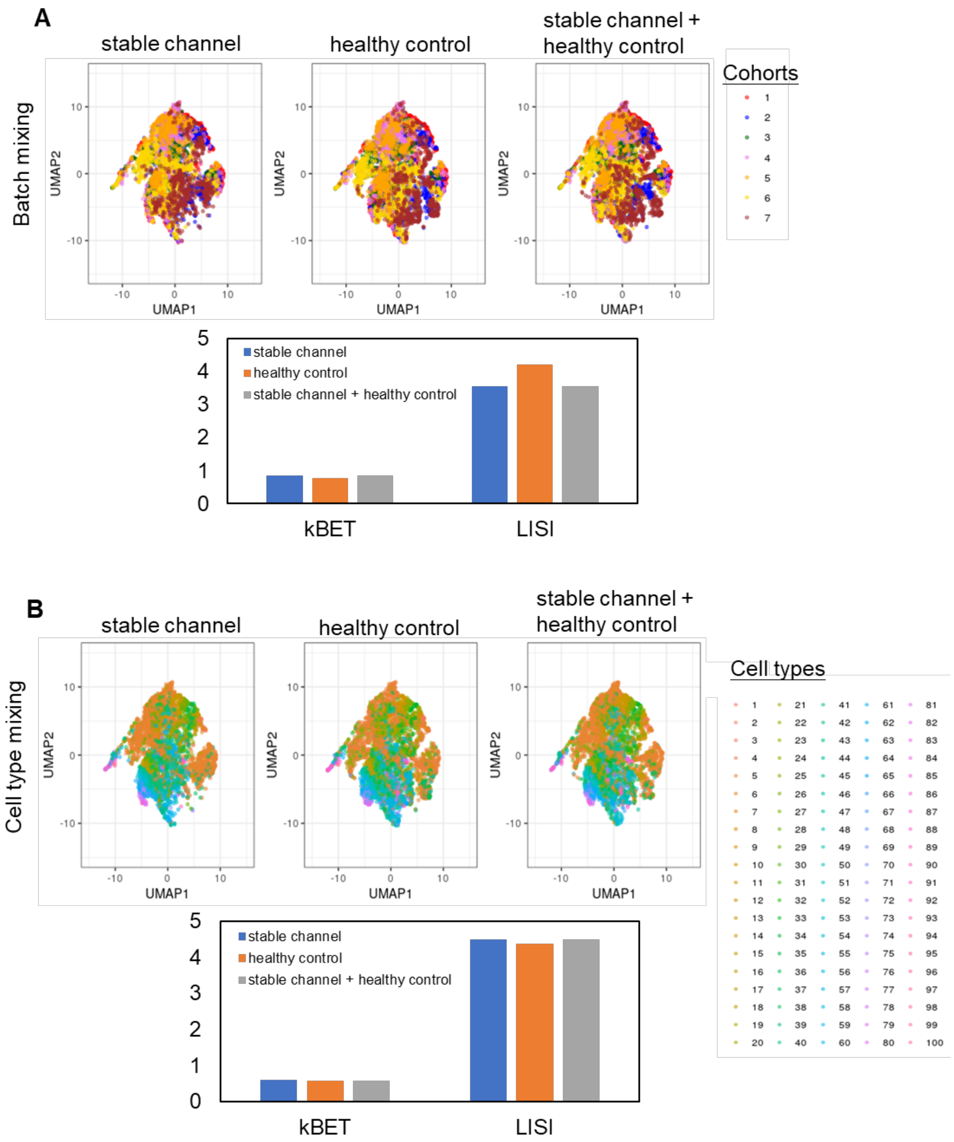

Supplementary Figure 15. Comparison of batch and cell mixing effects of combined healthy control samples normalized by healthy control anchors and stable channels. The degree of (A) batch mixing and (B) cell type mixing of combined healthy control samples after CytofIn normalization using healthy control, stable channels or combination of healthy control anchor and stable channels as evaluated by the kBET and LISI index. Similar kBET and LISI index values were observed using either approach and there was no additive effect when the two approaches were combined.

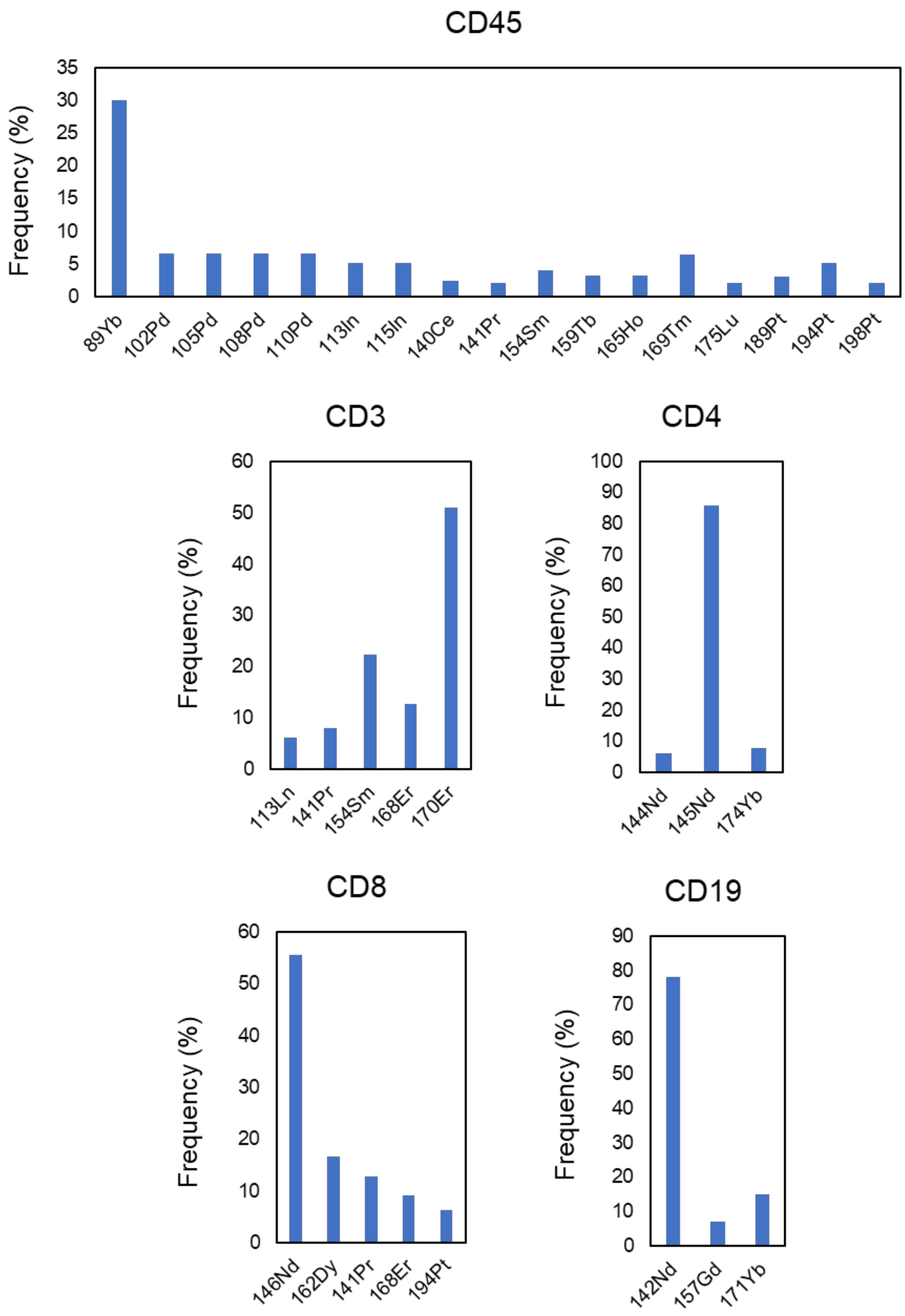

Supplementary Figure 16. Metal conjugations on the top five most frequent overlapping markers from Flow Repository. The percentage of the conjugated metals on the top five most frequent markers: CD45, CD3, CD4, CD8 amd CD19 from the FlowRepository retrieved with a PBMC tag. Note that each marker has a metal type that is predominantly used in the database.

A

Markers	ZY6C	Metal sensitivity	ZYDG	Metal sensitivity
CD19	Nd142	0.4	Nd142	0.4
CD8	Nd146	0.5	Er168	1
CD3	Sm154	0.8	Er170	0.9
CD4	Yb174	0.8	Nd145	0.5

Δ metal sensitivity: > 0.1 (green); < 0.1 (orange); 0 (yellow)

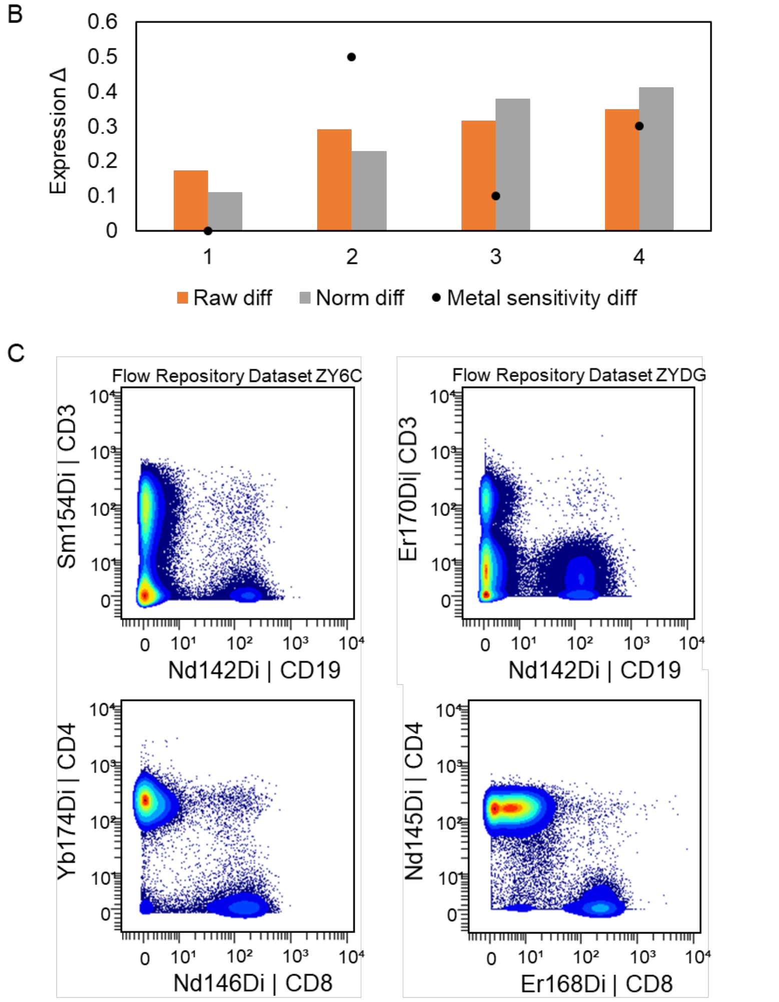

Supplementary Figure 17. Effects of conjugated metal sensitivity on marker signal for public dataset integration. (A) Metal sensitivity difference in four selected makers: CD19, CD8, CD3, and CD4 in our combined melanoma datasets were quantified as previously reported. (B) Expression difference of the four selected markers pre and post CytofIn normalization. (C) Biaxial plots of one representative patient samples selected from each of the two datasets based on the four markers demonstrate preservation of population distributions after normalization with CytofIn.

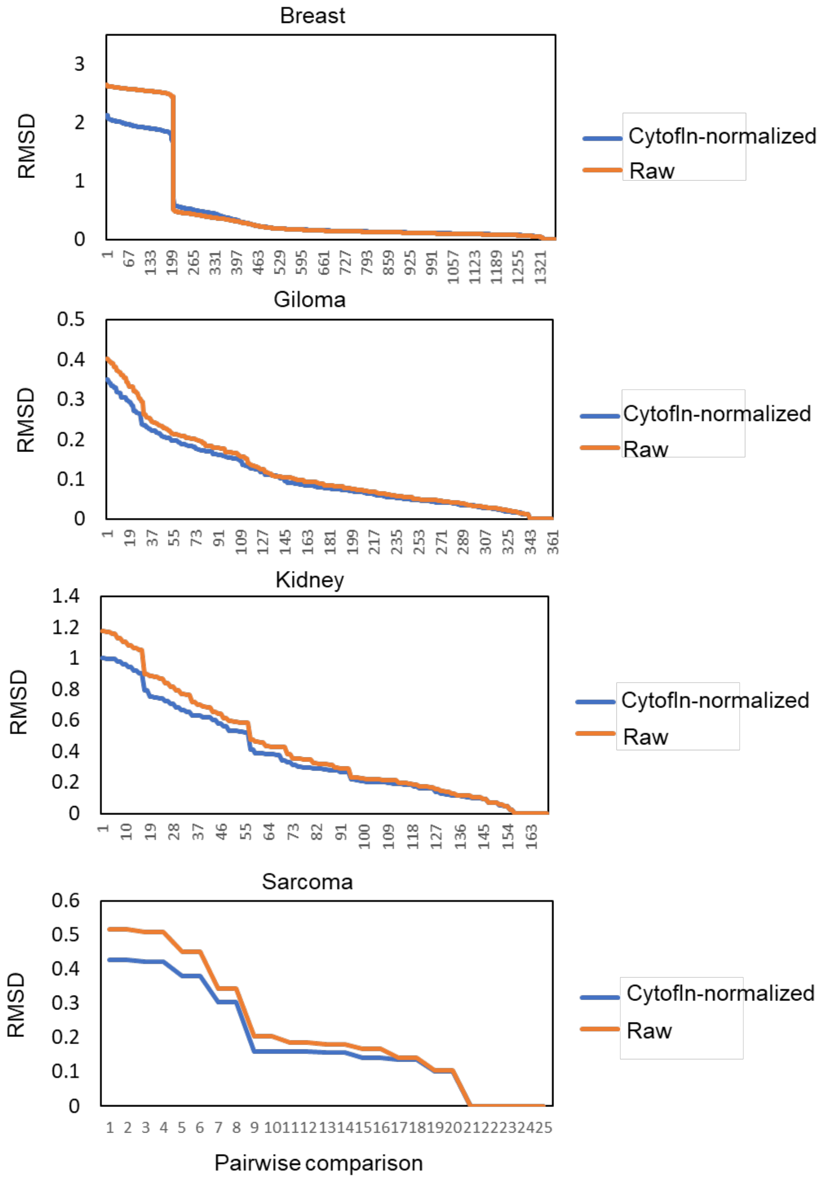

Supplementary Figure 18. Pair-wise RMSD evaluation of batch effects reduction in TIL cancer datasets from CytofIn integration. Pair-wise mean expression difference measured by RMSD values for each file between and within the TIL datasets from four cancer types: breast, glioma, kidney, and sarcoma. The x-axis represents the sample-pairs and y-axis represents pairwise RMSD values. Within the breast cancer samples, these were evaluated within and between the two independent TIL datasets. Giloma, Kidney and Sarcoma were evaluated within the same TIL dataset.

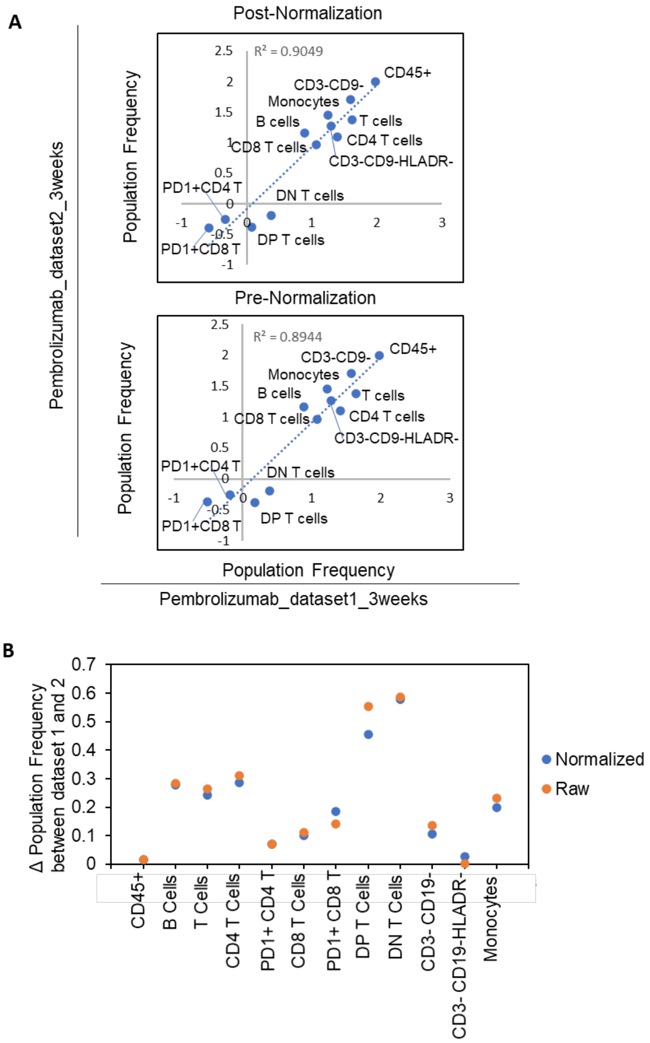

Supplementary Figure 19. Comparison of batch-normalized melanoma PBMC patient samples under anti-PD treatment from two independent datasets. (A) Correlation analysis of T cell subpopulation frequencies between batch-normalized melanoma PBMC samples treated by Pembrolizumab at the three-week time point from Wei *et al.* (dataset 2) and Greenplate *et al.* (dataset 1). Note that the correlation (R^2 values) increased from 0.89 to 0.91 post-batch normalization between the two datasets. (B) The difference in the subpopulation frequency between the two datasets pre- and post batch normalization. Note that the average RMSD values decrease from 9 to 7.95 post batch normalization.

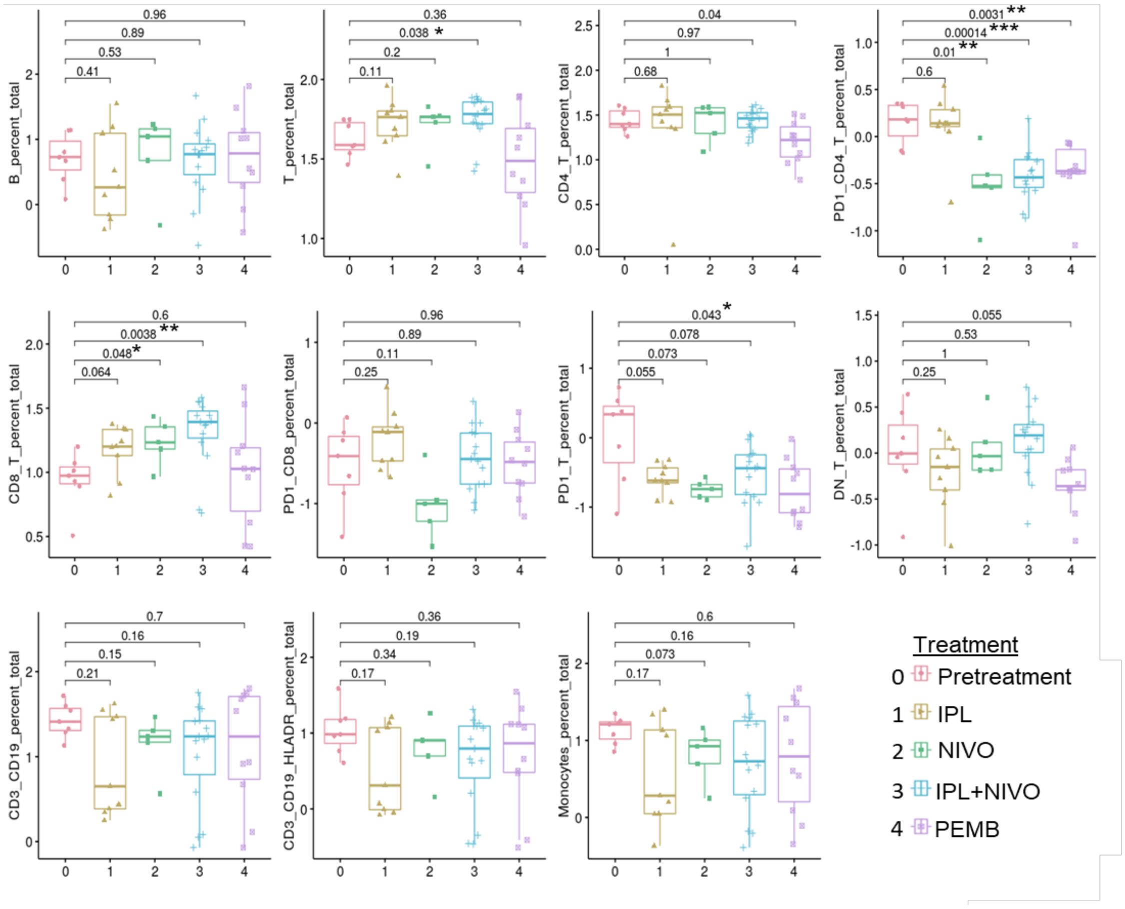

Supplementary Figure 20. Subpopulation frequency of immune cells from the PBMC samples of melanoma patients batch-normalized between two melanoma datasets. PBMCs from melanoma patients consisting of pretreatment samples (n=7) from dataset 1and Ipilimumab (n=9), Nivolumab (n=5), the combination of Ipilimumab and Nivolumab (n=15), and Pembrolizumab-treated (n=10) samples from dataset 2 were gated for T cell subpopulations using a common gating strategy as described by Greenplate *et al*. The band indicates the median, the box indicates the first and third quartiles and the whiskers indicate ± 1.5 × interquartile range. The population frequencies under each immunotherapy were compared to that of pretreatment samples using a two-sided Wilcoxon test (p-values: *<0.05, **<0.01, ***<0.001).

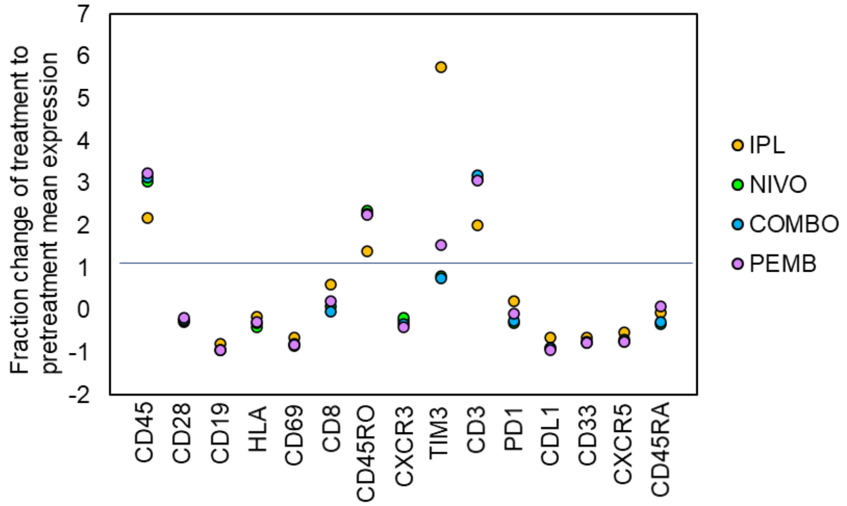

Supplementary Figure 21. Effects of immunotherapies on the protein expression of immune subpopulations from PBMC samples of melanoma patients. Fraction mean expression changes from $PD1^+CD4^+$ subpopulations treated with Ipilimumab (n=9), Nivolumab (n=5), a combination of Nivolumab and Ipilimumab (n=15), and Pembrolizumab (n=10) to pretreatment PBMC samples (n=7) of melanoma patients by integrated analysis of two melanoma datasets.

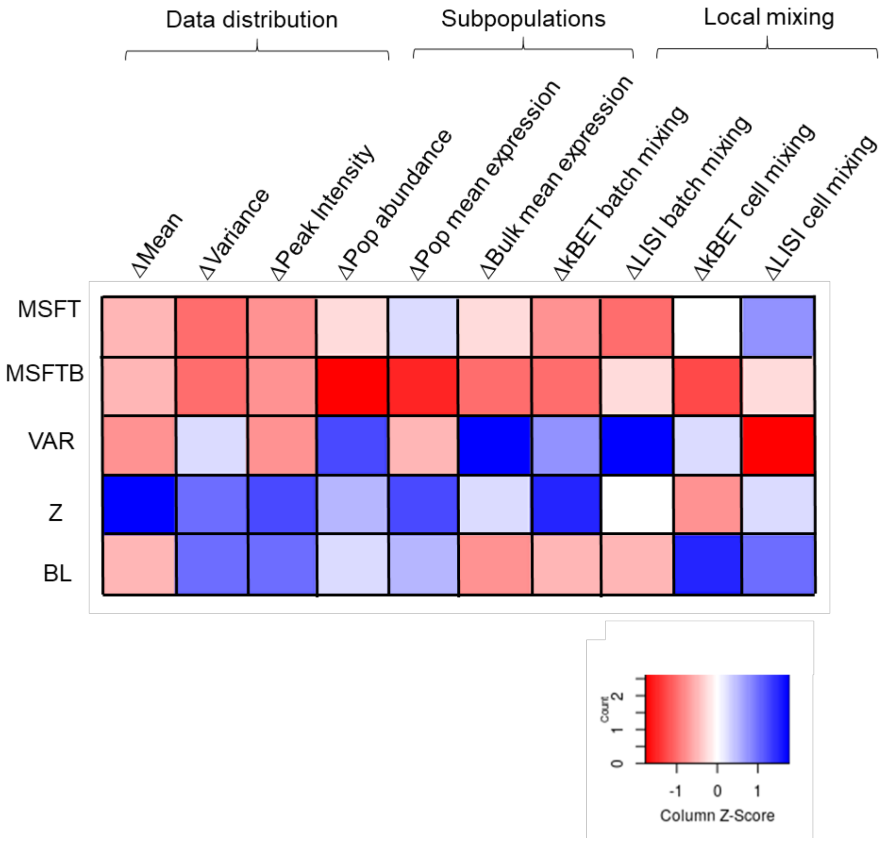

Supplementary Figure 22. Performance summary of five normalization functions in CytofIn. The heatmap summarizes the changes of 10 metrics between bead and batch normalization by five CytofIn normalization functions. The metrics include changes in data distribution (mean, variance, peak intensity), subpopulation features (abundance and mean expression) as well as degree of local batch and cell type mixing based on kBET or LISI metrics.

Supplementary Tables

Supplementary Table 1. Comparison of available batch normalization methods for mass cytometry datasets. The ability to perform panel alignment, the requirement of identical standards, and re-normalization with new data were compared between CytofIn and other published batch normalization approaches for mass cytometry data.

Batch Normalization Methods	Performs Panel Alignment	Requires Identical Standards	Requires renormalization with new data
**Ideal**	**Y**	**N**	**N**
**CytofIn**	Y	N	N
**BatchAdjust (Schuyler,2019)**	N	Y	N
**CyTOF RUV (Trussart, 2020)**	N	Y	N
**CytoNorm (Van Gassen,2019)**	N	Y	N
**Quantile (Good, 2019)**	N	N	Y
**Bead (Finck, 2013)**	N	Y	Y

Supplementary Table 2. Antibody Panel for the CyTOF Leukemia Dataset.

Metal	Protein	Clone	Manufacturer	Catalog Number	Final concentration (ug/mL)	Localization	Panel
(In113)Di	CD235	HIR2**	Biolegend	306602	2	Surface	all
(In113)Di	CD61	VI-PL2**	BD	306402	1	Surface	all
(In115)Di	CD45	HI30	Biolegend	304002	2	Surface	all
(La139)Di	cPARP	F21-852	BD	552597	1.5	Intracellular	all
(Pr141)Di	pPLCg2	K86-689.37	BD	custom	1	Surface	all
(Nd142)Di	CD19	H1B19	Biolegend	302202	2	Surface	all
(Nd143)Di	CD22	HIB22	Biolegend	302502	2	Surface	all
(Nd144)Di	p4EBP1	236B4	Cell Signaling Technology	2855BF	1	Intracellular	all
(Nd145)Di	tIkaros	D10E5	Cell Signaling Technology	9034BF	4	Intracellular	all
(Nd146)Di	CD79b	CB3-1	Biolegend	555678	6	Surface	all
(Sm147)Di	CD20	2H7	Biolegend	302302	2	Surface	all
(Nd148)Di	CD34	8G12	Biolegend	343502	1	Surface	all
(Sm149)Di	CD179a	HSL96	Biolegend	347402	1.5	Intracellular	all
(Nd150)Di	pSTAT5	47	BD	custom	1	Intracellular	all
(Eu151)Di	CD123	6H6*	Biolegend	306002	2	Surface	1,2
(Eu151)Di	GCR	D8H2*	Cell Signaling Technology	3660BF	4	Intracellular	3
(Sm152)Di	Ki67	B56	BD	556003	2	Intracellular	all
(Eu153)Di	IgMi	polyclonal	Novus	NBP1-75017	1	Intracellular	all
(Sm154)Di	Kappa	MHK-49**	Biolegend	316502	1	Surface	all
(Sm154)Di	Lambda	MHL-38**	Biolegend	316502	1	Surface	all
(Gd155)Di	pIkaros	STA9*	Epitomics (custom)	custom	2	Intracellular	1,2
(Gd155)Di	BAD	Y208*	Abcam	ab220116	4	Intracellular	3
(Gd156)Di	CD10	HI10a	Biolegend	312202	1	Surface	all
(Gd157)Di	CD132	TUGh4*	R&D Systems	AF284	4	Surface	2
(Gd158)Di	CD179b	HSL11	Biolegend	349802	1	Intracellular	all
(Tb159)Di	pAkt	193H12*	Cell Signaling Technology	4060BF	1	Intracellular	1,2
(Tb159)Di	BCL2	100*	Biolegend	658702	1	Intracellular	3
(Gd160)Di	CD24	ML5	Biolegend	311102	2	Surface	all
(Dy161)Di	TSLPr	eBio1A6	eBioscience	eBio1A6	2	Surface	all
(Dy162)Di	CD127	HCD127	Biolegend	351302	1	Surface	all
(Dy163)Di	RAG1	D36B3	Cell Signaling Technology	3968BF	2	Intracellular	all
(Dy164)Di	TdT	E17-1519	BD	custom	2	Intracellular	all
(Ho165)Di	Pax5	1H9	eBioscience	649702	1	Intracellular	all
(Er166)Di	pSyk	17a	BD	custom	2	Intracellular	all
(Er167)Di	CD43	CD43-10G7	Biolegend	343202	2	Surface	all
(Er168)Di	CD38	HIT2	Biolegend	303502	2	Surface	all
(Tm169)Di	CD58	TS2-9*	Biolegend	330902	2	Surface	1,2
(Tm169)Di	preBCR	HSL2*	Biolegend	custom	1.5	Surface	3
(Er170)Di	CD3	HIT3a	Biolegend	300302	0.5	Surface	all
FITC	CD33	HIM3-4**	Biolegend	303304	20	Surface	1,2
FITC	CD16	3G8**	Biolegend	302006	20	Surface	1,2
(Yb171)Di	FITC	FIT-22	Biolegend	408302	2	Intracellular	1,2
(Yb171)Di	XIAP	polyclonal*	R&D Systems	AF8221	2	Intracellular	3
(Yb172)Di	pS6	N7-548	BD	custom	2	Intracellular	all

(Yb173)Di	pErk	D13.14.4E	Cell Signaling Technology	4370BF	0.5	Intracellular	all
(Yb174)Di	HLADR	L243	Biolegend	307602	2	Surface	all
(Lu175)Di	IgMs	polyclonal	Novus	NBP1-75017	1	Surface	all
(Yb176)Di	pCreb	87G3	Cell Signaling Technology	9198BF	0.5	Intracellular	all
(Bi209)Di	CD33	WM53*	Biolegend	303419	1	Surface	3
(Bi209)Di	CD16	3G8*	Biolegend	302002	0.5	Surface	3

Supplementary Table 3. Quantitation of Changes in Original Data Distribution by CytofIn Batch Normalization Functions.

Transformation	Δmean	Δvariance	Δpeak intensity
**MSFT**	0.1 ± 0.04	0	0.1 ± 0.02
**MSFTB**	0.1 ± 1.92e-08	0	0.1 ± 3.43e-8
**BL**	0.05 ± 0.07	0.15 ± 0.19	0.52 ± 0.1
**VAR**	0.45 ± 0.45	0.23 ± 0.12	4.48 ± 4.95
**Z**	0.1 ± 0.04	0.23 ± 0.12	4.14 ± 4.42

Supplementary Table 4. Quantitation of Changes in Subpopulation Features from Bead Normalization by CytofIn Batch Normalization Functions.

Transformation	Δbead-normalized population abundance	Δbead-normalized marker expression
**MSFT**	1.09±1.59	0.37±0.29
**MSFTB**	0.73±0.82	0.17±0.13
**BL**	1.43±1.77	0.28±0.24
**VAR**	1.26±1.3	0.52±0.44
**Z**	1.23±1.75	0.41±0.39
