# Supplementary material for: CytofIn enables integrated analysis of public mass cytometry datasets using generalized anchors
Source: Nat Commun. 2022 Feb 17;13:934. doi: 10.1038/s41467-022-28484-5 (PMC8854441; doi:10.1038/s41467-022-28484-5)
Supplement: Supplementary file 2 — Description of Additional Supplementary Files [file 41467_2022_28484_MOESM2_ESM.pdf]

Description of Additional Supplementary Files

File Name: Supplementary Data 1

Description: Metadata Table for the CyTOF Leukemia Dataset: File Names and Conditions

File Name: Supplementary Data 2

Description: Homogenized panel used in the CyTOF Leukemia normalization.

File Name: Supplementary Data 3

Description: PBMC Files of Melanoma Datasets.
