# Supplementary material for: CytofIn enables integrated analysis of public mass cytometry datasets using generalized anchors
Source: Nat Commun. 2022 Feb 17;13:934. doi: 10.1038/s41467-022-28484-5 (PMC8854441; doi:10.1038/s41467-022-28484-5)
Supplement: Supplementary file 6 — Reporting Summary [file 41467_2022_28484_MOESM6_ESM.pdf]

nature portfolio

Corresponding author(s): Kara L. Davis

Last updated by author(s): Jan 27, 2022

Reporting Summary

Nature Portfolio wishes to improve the reproducibility of the work that we publish. This form provides structure for consistency and transparency in reporting. For further information on Nature Portfolio policies, see our Editorial Policies and the Editorial Policy Checklist.

Statistics

For all statistical analyses, confirm that the following items are present in the figure legend, table legend, main text, or Methods section.

n/a	Confirmed	
☐	☒	The exact sample size (*n*) for each experimental group/condition, given as a discrete number and unit of measurement
☐	☒	A statement on whether measurements were taken from distinct samples or whether the same sample was measured repeatedly
☐	☒	The statistical test(s) used AND whether they are one- or two-sided<br>*Only common tests should be described solely by name; describe more complex techniques in the Methods section.*
☐	☒	A description of all covariates tested
☒	☐	A description of any assumptions or corrections, such as tests of normality and adjustment for multiple comparisons
☐	☒	A full description of the statistical parameters including central tendency (e.g. means) or other basic estimates (e.g. regression coefficient) AND variation (e.g. standard deviation) or associated estimates of uncertainty (e.g. confidence intervals)
☐	☒	For null hypothesis testing, the test statistic (e.g. *F*, *t*, *r*) with confidence intervals, effect sizes, degrees of freedom and *P* value noted<br>*Give P values as exact values whenever suitable.*
☒	☐	For Bayesian analysis, information on the choice of priors and Markov chain Monte Carlo settings
☒	☐	For hierarchical and complex designs, identification of the appropriate level for tests and full reporting of outcomes
☐	☒	Estimates of effect sizes (e.g. Cohen's *d*, Pearson's *r*), indicating how they were calculated

Our web collection on statistics for biologists contains articles on many of the points above.

Software and code

Policy information about availability of computer code

Data collection	The mass cytometry data were extracted and processed using the Fludigm CyTOF software (version 7.0).
Data analysis	The mass cytometry data were analyzed using the following packages from the R bioconductor software (version 2.2): flowcore, ggcyto, openCyto and flowWorkspace. Network and statistical analysis were performed using the Cytoscape software (version 3.8.0) and the R statistical software (version 3.6.3). Benchmark study were conducted using the CytoNorm, CytoRUV and Seurat (version 4.0.2) software packages and kBET, LISI and FlowSOM R packages. Data integration and normalization was performed using the CytofIn R package (https://github.com/bennyyclo/Cytofin/).

For manuscripts utilizing custom algorithms or software that are central to the research but not yet described in published literature, software must be made available to editors and reviewers. We strongly encourage code deposition in a community repository (e.g. GitHub). See the Nature Portfolio guidelines for submitting code & software for further information.

Data

Policy information about availability of data

All manuscripts must include a data availability statement. This statement should provide the following information, where applicable:

- Accession codes, unique identifiers, or web links for publicly available datasets
- A description of any restrictions on data availability
- For clinical datasets or third party data, please ensure that the statement adheres to our policy

The data that supports the findings and generated during the current study are available as source data file located at https://doi.org/10.5281/zenodo.5911417 and the Flow Repository (http://flowrepository.org/).

Field-specific reporting

Please select the one below that is the best fit for your research. If you are not sure, read the appropriate sections before making your selection.

- [x] Life sciences
- [ ] Behavioural & social sciences
- [ ] Ecological, evolutionary & environmental sciences

For a reference copy of the document with all sections, see nature.com/documents/nr-reporting-summary-flat.pdf

Life sciences study design

All studies must disclose on these points even when the disclosure is negative.

Sample size	Sample size were determined by the number of patients from each clinical study. We analyzed 7 B-cell acute lymphoblastic leukemia cohorts for a total of 989 samples. The large sample size were chosen to provide a robust validation and analysis of our study.
Data exclusions	No data were excluded from the analyses.
Replication	Replicates of the primary samples were not routinely performed but sometimes included in these datasets.
Randomization	Primary patient sample allocation was based on the cohort group from each clinical study. The order of the cohort group and/or patient sample does not impact our analysis. Therefore, randomization is not required in our study.
Blinding	Primary patient sample allocation was based on the cohort group from each clinical study and the label assignment is required to investigate batch effect. Therefore, blinding is not required in our analysis.

Reporting for specific materials, systems and methods

We require information from authors about some types of materials, experimental systems and methods used in many studies. Here, indicate whether each material, system or method listed is relevant to your study. If you are not sure if a list item applies to your research, read the appropriate section before selecting a response.

Materials & experimental systems

n/a	Involved in the study	
☐	☒	Antibodies
☒	☐	Eukaryotic cell lines
☒	☐	Palaeontology and archaeology
☒	☐	Animals and other organisms
☐	☒	Human research participants
☒	☐	Clinical data
☒	☐	Dual use research of concern

Methods

n/a	Involved in the study	
☒	☐	ChIP-seq
☒	☐	Flow cytometry
☒	☐	MRI-based neuroimaging

Antibodies

Antibodies used	Please see Supplementary Table 2
Validation	All mass cytometry antibodies have been validated in our laboratory as a part of routine development process using positive and negative control or as previously published.

Human research participants

Policy information about studies involving human research participants

Population characteristics	De-identified samples from children and adults with B cell acute lymphoblastic leukemia or B cell lymphoma were utilized in this study.
Recruitment	Samples were collected as part of IRB-approved clinical research studies.
Ethics oversight	The Institutional Review Board (IRB) of Stanford University, University Milano-Biococa or St. Jude Children's Research Hospital approved the use of these primary samples for this work.

Note that full information on the approval of the study protocol must also be provided in the manuscript.
